# LINC00982-encoded protein PRDM16-DT regulates *CHEK2* splicing to suppress colorectal cancer metastasis and chemoresistance

**DOI:** 10.7150/thno.95485

**Published:** 2024-05-27

**Authors:** Hui-Fang Hu, Lei Han, Jia-Ying Fu, Xuan He, Ji-Feng Tan, Qing-Ping Chen, Jing-Ru Han, Qing-Yu He

**Affiliations:** 1 MOE Key Laboratory of Tumor Molecular Biology and State Key Laboratory of Bioactive Molecules and Druggability Assessment, College of Life Science and Technology, Jinan University, Guangzhou 510632, China.; 2 Biomedicine Research and Development Center, National Engineering Research Center of Genetic Medicine, Jinan University, Guangzhou, Guangdong 510632, China.; 3 The First-Affiliated Hospital, Jinan University, Guangzhou, Guangdong 510632, China.

**Keywords:** Colorectal cancer, PRDM16-DT, Fibroblasts, Cimicifugoside H-1, Drug resistance.

## Abstract

Metastasis is one of the key factors of treatment failure in late-stage colorectal cancer (CRC). Metastatic CRC frequently develops resistance to chemotherapeutic agents. This study aimed to identify the novel regulators from "hidden" proteins encoded by long noncoding RNAs (lncRNAs) involved in tumor metastasis and chemoresistance.

**Methods:** CRISPR/Cas9 library functional screening was employed to identify the critical suppressor of cancer metastasis in highly invasive CRC models. Western blotting, immunofluorescence staining, invasion, migration, wound healing, WST-1, colony formation, gain- and loss-of-function experiments, *in vivo* experimental metastasis models, multiplex immunohistochemical staining, immunohistochemistry, qRT-PCR, and RT-PCR were used to assess the functional and clinical significance of FOXP3, PRDM16-DT, HNRNPA2B1, and L-*CHEK2*. RNA-sequencing, co-immunoprecipitation, qRT-PCR, RT-PCR, RNA affinity purification, RNA immunoprecipitation, MeRIP-quantitative PCR, fluorescence *in situ* hybridization, chromatin immunoprecipitation and luciferase reporter assay were performed to gain mechanistic insights into the role of PRDM16-DT in cancer metastasis and chemoresistance. An oxaliplatin-resistant CRC cell line was established by *in vivo* selection. WST-1, colony formation, invasion, migration, Biacore technology, gain- and loss-of-function experiments and an *in vivo* experimental metastasis model were used to determine the function and mechanism of cimicifugoside H-1 in CRC.

**Results:** The novel protein PRDM16-DT, encoded by LINC00982, was identified as a cancer metastasis and chemoresistance suppressor. The down-regulated level of PRDM16-DT was positively associated with malignant phenotypes and poor prognosis of CRC patients. Transcriptionally regulated by FOXP3, PRDM16-DT directly interacted with HNRNPA2B1 and competitively decreased HNRNPA2B1 binding to exon 9 of *CHEK2*, resulting in the formation of long *CHEK2* (L-*CHEK2*), subsequently promoting E-cadherin secretion. PRDM16-DT-induced E-cadherin secretion inhibited fibroblast activation, which in turn suppressed CRC metastasis by decreasing MMP9 secretion. Cimicifugoside H-1, a natural compound, can bind to LEU89, HIS91, and LEU92 of FOXP3 and significantly upregulated PRDM16-DT expression to repress CRC metastasis and reverse oxaliplatin resistance.

**Conclusions:** lncRNA LINC00982 can express a new protein PRDM16-DT to function as a novel regulator in cancer metastasis and drug resistance of CRC. Cimicifugoside H-1 can act on the upstream of the PRDM16-DT signaling pathway to alleviate cancer chemoresistance.

## Introduction

Colorectal cancer (CRC) is one of the most common malignant tumors, ranking second in women and third in men worldwide 1. The incidence of CRC is rising rapidly in many low- and middle-income countries 2. Approximately 45% of newly diagnosed CRC patients develop metastasis due to the tumor aggressiveness 3. Currently, chemotherapy efficacy is limited due to severe adverse effects, drug resistance, and low efficacy on distant metastatic cancer cells. Drug resistance and cancer metastasis are two interrelated phenomena in cancer progression 4. Cancer metastasis significantly contributes to drug resistance in CRC 5. Thus, there is an urgent need to investigate critical regulators involved in the molecular mechanisms of cancer metastasis and to explore effective and safe agents for CRC therapy.

Transcripts of more than 200 nt lacking protein-coding ability are long-noncoding RNAs (lncRNAs). Many studies revealed that lncRNAs, acting as oncogenes or tumor suppressors, have significant functions in tumorigenesis and cancer progression, including proliferation, differentiation, and metastasis 6, 7, 8. With advances in deep-sequencing technologies over the last decade, a large number of previously unknown lncRNAs were identified 9 that can encode proteins or peptides contributing to the development of cancers. For example, LINC00665-206 (NR_038278.1), a transcript of LINC00665, encodes a peptide CIP2A-BP, which mediates breast cancer metastasis by inhibiting the PI3K/AKT/NF-kB signaling pathway 10. Nevertheless, protein/peptide-encoding properties and biological functions of most of these lncRNAs remain to be characterized.

Using full-length mRNA sequencing, ribosome profiling, shotgun proteomics, and immunoblotting, we previously reported 314 lncRNAs encoding new proteins with more than 50 amino acids 11, representing an undiscovered human proteome with completely unknown biological functions. We hypothesized that the proteins encoded by 314 lncRNAs contain unknown regulators associated with drug resistance and cancer metastasis. We used CRISPR/Cas9 functional screening of the 314 lncRNAs in highly invasive CRC cells. We identified and characterized LINC00982, which could encode a new protein, PRDM16-DT, and significantly inhibit CRC metastasis.

RNA alternative splicing (AS) is a critical process that generates structurally and functionally distinct mRNA and protein variants to expand the diversity of metazoan genomes 12. A pre-mRNA is produced during gene transcription and subjected to diverse splicing events, generating a large number of spliced variants due to alternative 3' (A3SSs) and 5' splice sites (A5SSs), exon skipping (SE), mutually exclusive exons (MXEs), and intron retention (IR) in many genes 13. Increasing evidence shows that AS controls various hallmarks of cancer 14. For example, hnRNPA1-dependent *PKM* splicing regulated by HOXB-AS3 peptide induces colon cancer growth 10. Also, dysregulated N6-methyladenosine (m6A) modification has been shown to be associated with various cancers 15, and the presence of m6A on transcripts may affect AS 16. The RNA-binding protein HNRNPA2B1, a splicing regulator known as an m6A “reader”, can also bind to m6A-bearing RNAs 17. However, the significance of AS and RNA m6A modification in cancer development remains poorly understood.

In our continuing efforts to functionally characterize the unexplored proteins, this study discovered that LINC00982, with a noncoding number NR_015440.3.4, could express an anticancer protein PRDM16-DT. The biological function, mechanism, and clinical significance of PRDM16-DT were comprehensively investigated *in vitro* and *in vivo*. Our findings demonstrated that PRDM16-DT regulated the RNA splicing of *CHEK2*, stimulating E-cadherin expression to inhibit the activation of fibroblasts, subsequently suppressing CRC metastasis. We have also shown that HNRNPA2B1-mediated AS is directly involved in PRDM16-DT-regulated cancer metastasis. We further identified that the natural compound cimicifugoside H-1 could upregulate PRDM16-DT via the upstream transcription factor FOXP3, suppressing cancer metastasis and reversing oxaliplatin resistance of CRC.

## Results

### Identification of key drivers of colon tumorigenesis and chemoresistance

We investigated the key signaling pathways and biological processes involved in CRC progression and drug resistance by analyzing the National Center for Biotechnology Information Gene Expression Omnibus (GEO) dataset GSE52056, which included 23 CRC and paired normal tissues (Figure S1A). The differentially expressed genes (DEGs, fold-change ≥ 1.5, p-value ≤ 0.05, Figure S1B) were analyzed by Kyoto Encyclopedia of Genes and Genomes (KEGG) analysis. The results revealed that pathways associated with cancer metastasis, including tight junctions, gap junctions, and ECM receptor interactions, were key pathways in CRC progression (Figure S1C). The GEO dataset GSE69657, including 13 cases of chemotherapy-sensitive and 17 cases of chemotherapy-resistant CRC tissues (Figure S1D), was used to identify DEGs involved in the chemoresistance of CRC (Figure S1D-E). Subsequently, the DEGs were analyzed by KEGG analysis showing that pathways associated with cancer metastasis, including cell adhesion, ECM receptor interaction, and focal adhesion, were key pathways in CRC chemoresistance (Figure S1F). We also analyzed The Cancer Genome Atlas (TCGA) dataset, which included 131 CRC tissues with chemotherapy. Based on the regulator expression levels of epithelial-mesenchymal transition (EMT), a crucial progression in cancer metastasis, CRC patients were clustered into two subtypes, cluster1 (C1, n = 33) and cluster2 (C2, n = 98) (Figure S1G-I). Kaplan-Meier survival analysis revealed significantly shorter survival of C1 than C2 (Figure S1J), suggesting that EMT plays a key role in chemoresistance of CRC.

Chemoresistance, such as oxaliplatin resistance of CRC, frequently leads to poor prognosis of CRC patients. Thus, we established an oxaliplatin resistant HCT116 cell line (HCT116-Luc-OR) through intravenous injection and oral administration of oxaliplatin (Figure S1K). As shown in Figure S1L-M, HCT116-Luc-OR cells were highly resistant to oxaliplatin for cell growth (IC50: 34.7 μM), invasion, and migration. OR cells also demonstrated stronger invasive and metastatic abilities than their parental cells, supporting the tight link between cancer metastasis and chemoresistance. Data-independent acquisition (DIA)-based proteomics was performed to identify the differentially expressed proteins (DEPs) in OR cells (HCT116-Luc vs HCT116-Luc-OR). Among the 7657 identified proteins (Figure S1N), 2121 were significantly regulated in OR cells, including 988 upregulated and 1133 downregulated proteins (fold-change ≥ 1.5, P ≤ 0.05; Table S2). Consistently, KEGG analysis showed that pathways associated with cancer metastasis also play an essential role in oxaliplatin resistance of CRC (Figure S1O). These results suggest that common pathways were involved in cancer metastasis and chemoresistance, we then proceeded to identify novel key regulators that could simultaneously regulate metastasis and chemoresistance.

### Protein PRDM16-DT encoded by LINC00982 is identified as a cancer metastasis suppressor

**Figure 1:**
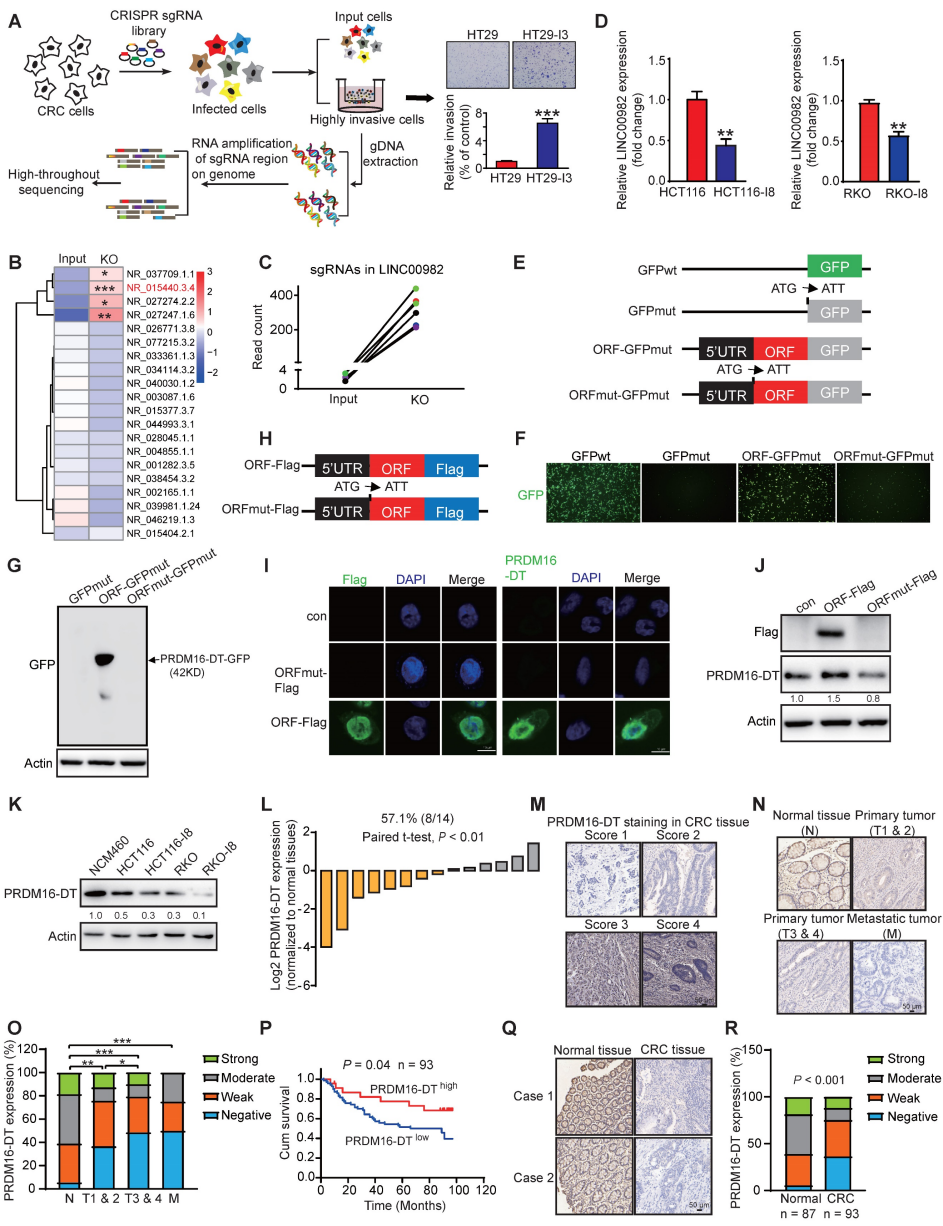
** PRDM16-DT encoded by LINC00982 is identified as a cancer metastasis suppressor and is of clinical significance in CRC. (A)** Schematic diagram of CRISPR/Cas9 screening **(B)** Heatmap of lncRNAs involved in cancer metastasis. **(C)** Read counts of LINC00982 sgRNAs are significantly increased in sgRNA-transduced cells (KO) as compared to input cells. **(D)** QRT-PCR assay displays markedly decreased LINC00982 expression in I8 cells compared to their parental cells. n = 3/experiments. **(E)** Structure of the PRDM16-DT-GFP fusion plasmids. Mut means ATG (start codon) is mutated to ATT. **(F-G)** PRDM16-DT-GFP fusion protein in ORF-GFPmut-transfected cells using GFP fluorescence **(F)** and Western blotting **(G)**. n = 3/experiments. (H) Structure of the Flag fusion plasmids. Mut means ATG (start codon) is mutated to ATT. **(I-J)** Using confocal microscopy **(I)** and Western blotting **(J)**, PRDM16-DT-Flag fusion protein expression is detected in ORF-Flag-transfected cells. n = 3/experiments. **(K)** Western blotting showing lower PRDM16-DT expression in CRC cells than NCM460 cells (normal colonic epithelial cells), and a further decrease in highly invasive I8 cells. n = 3/experiments. **(L)** Higher PRDM16-DT expression level in normal tissues than in CRC tissues (n = 14, P < 0.01). **(L)** Different staining scores for PRDM16-DT in CRC tissues. Scale bar, 50 μm. **(N-O)** IHC assay showing higher PRDM16-DT expression in most normal tissues than primary tumors (T1 & 2, T3 & 4) and metastatic tumors **(L)**. Scale bar, 50 μm. **(P)** Based on PRDM16-DT expression, Kaplan-Meier survival analysis shows a shorter survival in low PRDM16-DT-expressing patients than those with high PRDM16-DT expression (n = 93, P = 0.04). **(Q)** Representative staining results of PRDM16-DT expression in CRC and normal tissues. Scale bar, 50 μm. **(R)** Comparison of PRDM16-DT expression in 93 cases of CRC primary tumor tissues and 87 cases of adjacent normal tissues. Bars, SD; **, P < 0.01; ***, P < 0.001.

We previously reported that 314 lncRNAs in human cells may encode small proteins to form a hidden human proteome 11. To investigate whether any of these novel small proteins may play functional roles in cancer metastasis, we constructed a CRISPR/Cas9 library of sgRNAs targeting the 314 lncRNAs for functional screening using CRC metastasis as a model. The CRISPR/Cas9 library was transduced into HT29 cells. After 3 rounds of invasion selection (HT29-I3), gDNA from invaded and parental cells was extracted for PCR amplification, followed by high-throughput sequencing analysis (Figure 1A). The top 4 lncRNAs with significant functionality were selected in a pilot study to examine their effects on cancer cell invasion (Figure 1B). As shown in Figure S2, overexpressing the predicted ORF encoded by LINC00982 (NR_015440.3.4) most significantly repressed the invasion potential of CRC cells. When the start codon ATG of LINC00982 was mutated to ATT (ORFmut), the suppression effect on cell invasion was abolished, suggesting that LINC00982 functions as a coding RNA rather than a lncRNA. We focused on LINC00982 as it appeared to be a potential regulator of cancer invasion (P < 0.001, Figure 1B). In addition, the relative abundance of LINC00982-targeted sgRNAs was significantly increased in sgRNA-transduced cells (Figure 1C). And LINC00982 expression was significantly down-regulated in CRC tissues and in the highly metastatic CRC cell lines (HCT116-I8 and RKO-I8) that we established previously 18, as compared to normal tissues and parental cells (Figure 1D, S3A). These findings led us to further explore whether the protein encoded by LINC00982 functions as a novel regulator of CRC metastasis.

Bioinformatics analysis indicated the presence of 444-nucleotide ORF in LINC00982 with the potential to translate a 148-amino acid protein (Figure S3B). Since the LINC00982 is also known as PRDM16-DT for PRDM16 divergent transcript, we named the protein PRDM16-DT. To further ensure that the predicted ORF in LINC00982 could be encoded, we generated a series of plasmids in which GFPmut (with the start codon ATG mutated to ATT) was fused to the 5'UTR-ORF (ORF-GFPmut) and 5'UTR-ORFmut (ORFmut-GFPmut) (Figure 1E). As shown in Figure 1F, the PRDM16-DT-GFP fusion protein was observed in cells transfected with ORF-GFPmut- but not in ORFmut-GFPmut-transfected cells. Western blotting analysis using anti-GFP antibodies further confirmed that the PRDM16-DT-GFP fusion protein exhibited the predicted relative molecular mass in ORF-GFPmut-transfected cells but not in ORFmut-GFPmut-transfected cells (Figure 1G).

We also generated another series of constructs in which a Flag tag was fused to the 5'UTR-ORF (ORF-Flag) and the 5'UTR-ORFmut (ORFmut-Flag) (Figure 1H). The PRDM16-DT-Flag fusion protein was substantially expressed in ORF-Flag-transfected cells but not in ORFmut-Flag-transfected cells (Figure 1I). To further confirm the translation of the PRDM16-DT (ORF) in LINC00982, an antibody specific to PRDM16-DT was generated (Figure 1J).

The specificity of the antibody was characterized by establishing and subcutaneously injecting PRDM16-DT-deficient CRC cells (Figure S3C) into the flanks of nude mice. Tumor tissues were harvested after 2 weeks for immunohistochemistry (IHC). Staining was strong in the control group but extremely weak in PRDM16-DT-deficient tumor cells, suggesting good antibody specificity (Figure S3D). Similarly, as shown in Figure S3E, Western blotting assay demonstrated that increased PRDM16-DT-GFP fusion protein expression were detected using anti-PRDM16-DT antibodies. The addition of a GFP epitope to PRDM16-DT resulted in a significant shift in the mobility of PRDM16-DT band. Consistently, PRDM16-DT-Flag fusion protein expression was not detected by the anti-PRDM16-DT antibody when the start codon ATG of the predicted ORF in LINC00982 was mutated (Figure 1I-J). The PRDM16-DT protein was also identified by mass spectrometry (Figure S3F). Together, these data revealed that LINC00982 encodes PRDM16-DT protein with MW ~ 15KD.

### Clinical significance of PRDM16-DT expression in CRC

We detected a lower expression of PRDM16-DT in CRC cells than in normal colonic epithelial cells (NCM460), which further decreased in highly invasive I8 cells (Figure 1K). Meanwhile, the PRDM16-DT expression was stronger in normal tissues than in CRC tissues (Figure 1L). We then investigated the clinical significance of the PRDM16-DT expression using a microarray containing 93 CRC tumor tissues and 87 adjacent normal tissues (Figure 1M). As shown in Figure 1N-O, PRDM16-DT expression was higher in the majority of the normal tissues than in primary (T1 & 2, T3 & 4) and metastatic tumors (M), suggesting that PRDM16-DT expression is associated with tumor metastasis. Kaplan-Meier survival analysis revealed a significantly shorter survival in patients with low PRDM16-DT expression than those with high PRDM16-DT expression (P = 0.04, Figure 1P). In addition, most of the adjacent normal tissues had higher PRDM16-DT expression than tumor tissues (Figure 1Q-R). These data implicated PRDM16-DT as a tumor suppressor and potential prognostic biomarker in CRC.

### PRDM16-DT suppresses CRC metastasis and enhances the anticancer effects of oxaliplatin

**Figure 2:**
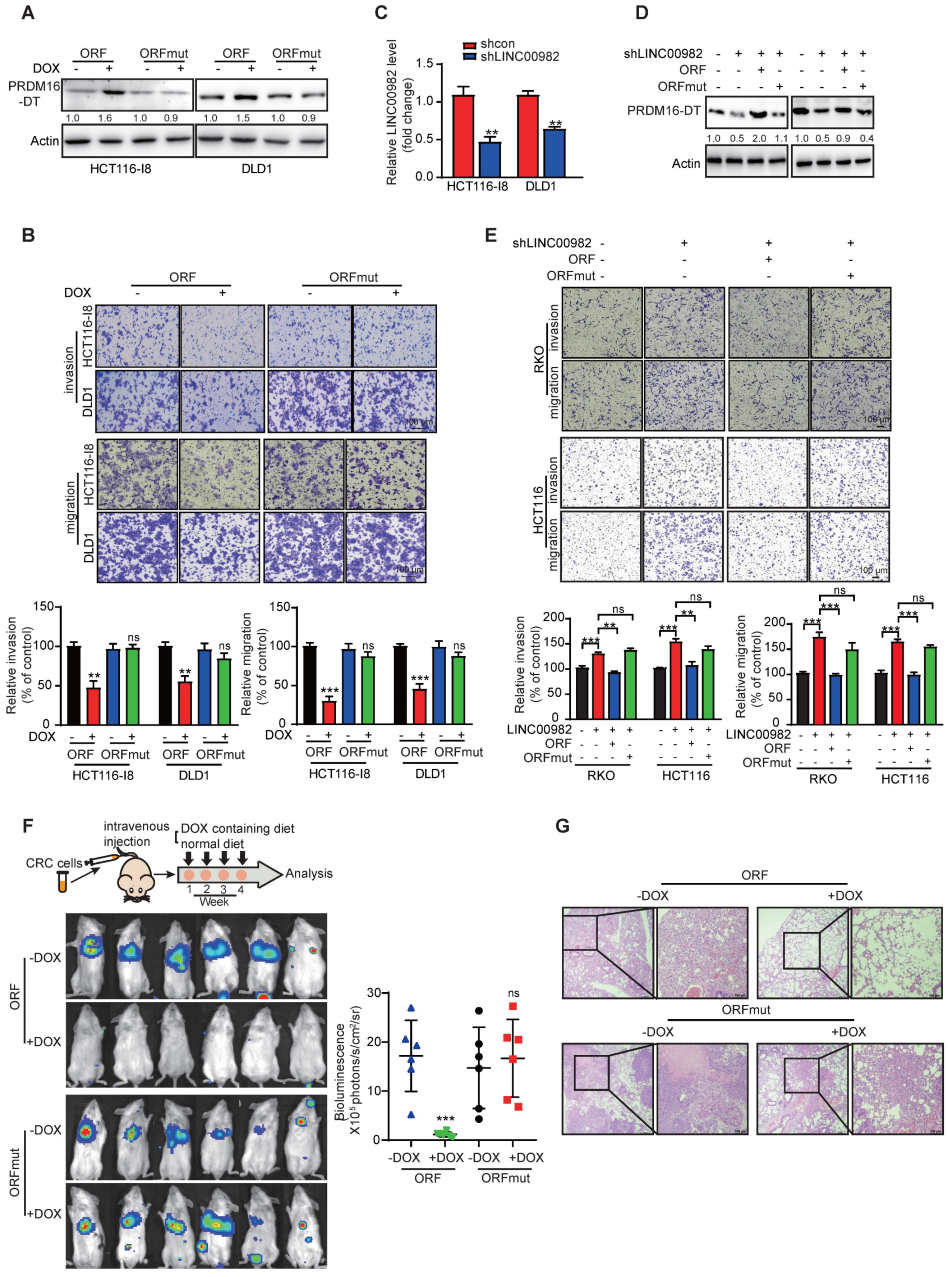
** PRDM16-DT inhibits CRC metastasis. (A)** Successful establishment of stable and doxycycline (DOX)-inducible ORF-overexpressing and ORFmut-overexpressing cell lines. Cells were treated with DOX (1 mg/mL) for 48 hours. n = 3/experiments. **(B)** PRDM16-DT significantly inhibits CRC migration and invasion ability. Cells were treated with DOX (1 mg/mL) for 48 h. n = 3/experiments. Scale bar, 100 μm. **(C)** Successful knockdown of LINC00982 in CRC cells. n = 3/experiments. **(D-E)** LINC00982 knockdown cells were transfected with ORF and ORFmut plasmids for 48 h and migration assay revealed that LINC00982 knockdown with shRNA sequence significantly promotes the CRC migration and invasion ability, restores expression with ORF, and significantly abrogates the promotion effect of silencing LINC00982. n = 3/experiments. Scale bar, 100 μm. **(F-G)** Bioluminescence imaging (n = 6 mice/group) **(F)**, and H&E staining (n = 3 mice/group) **(G)** show that PRDM16-DT reduces metastatic potential in mice. Mice were fed with either DOX containing or normal diet. Scale bar, 100 μm. Bars, SD; **, P < 0.01; ***, P < 0.001; ns, no significant difference.

We established stable doxycycline (DOX)-inducible ORF-overexpressing (PRDM16-DT protein) and ORFmut-overexpressing (PRDM16-DT lncRNA) cell lines to determine the biological function of PRDM16-DT in cancer metastasis and chemoresistance (Figure 2A). PRDM16-DT significantly reduced the migration and invasion ability of CRC (Figure 2B, S4A). Also, WST-1 and colony formation assays revealed that PRDM16-DT substantially enhanced the anticancer effects of oxaliplatin (Figure S4B-C). Conversely, knockout of LINC00982 with sgRNA markedly increased CRC migration and invasion, and restoring expression with ORF significantly abolished the effect of silencing LINC00982 (Figure S4D-E). These findings were also confirmed in PRDM16-DT knockdown cells with shRNA (Figure 2C-E, S4F). ORF re-expression also abolished the promotive effect of silencing LINC00982 on oxaliplatin resistance (Figure S4G-H). The re-expression of ORFmut did not produce the inhibitory effect since it did not encode the PRDM16-DT protein (Figure 2E, S4E-H).

Furthermore, we injected the stable and inducible ORF-overexpressing and ORFmut-overexpressing cell lines into the tail vein of mice to establish metastasis models. Mice were then randomized for treatment with DOX-containing or control diet. After four weeks, luciferase assays showed that PRDM16-DT (DOX-treated group) protein, rather than its lncRNA, suppressed cancer metastasis *in vivo* (Figure 2F), which was also confirmed by the histological analysis (Figure 2G). Collectively, these results indicated that LINC00982 encodes protein PRDM16-DT that functions as a cancer suppressor.

### FOXP3 transcriptionally regulates PRDM16-DT protein interacting with HNRNPA2B1 to suppress cancer metastasis and reverse oxaliplatin resistance

**Figure 3:**
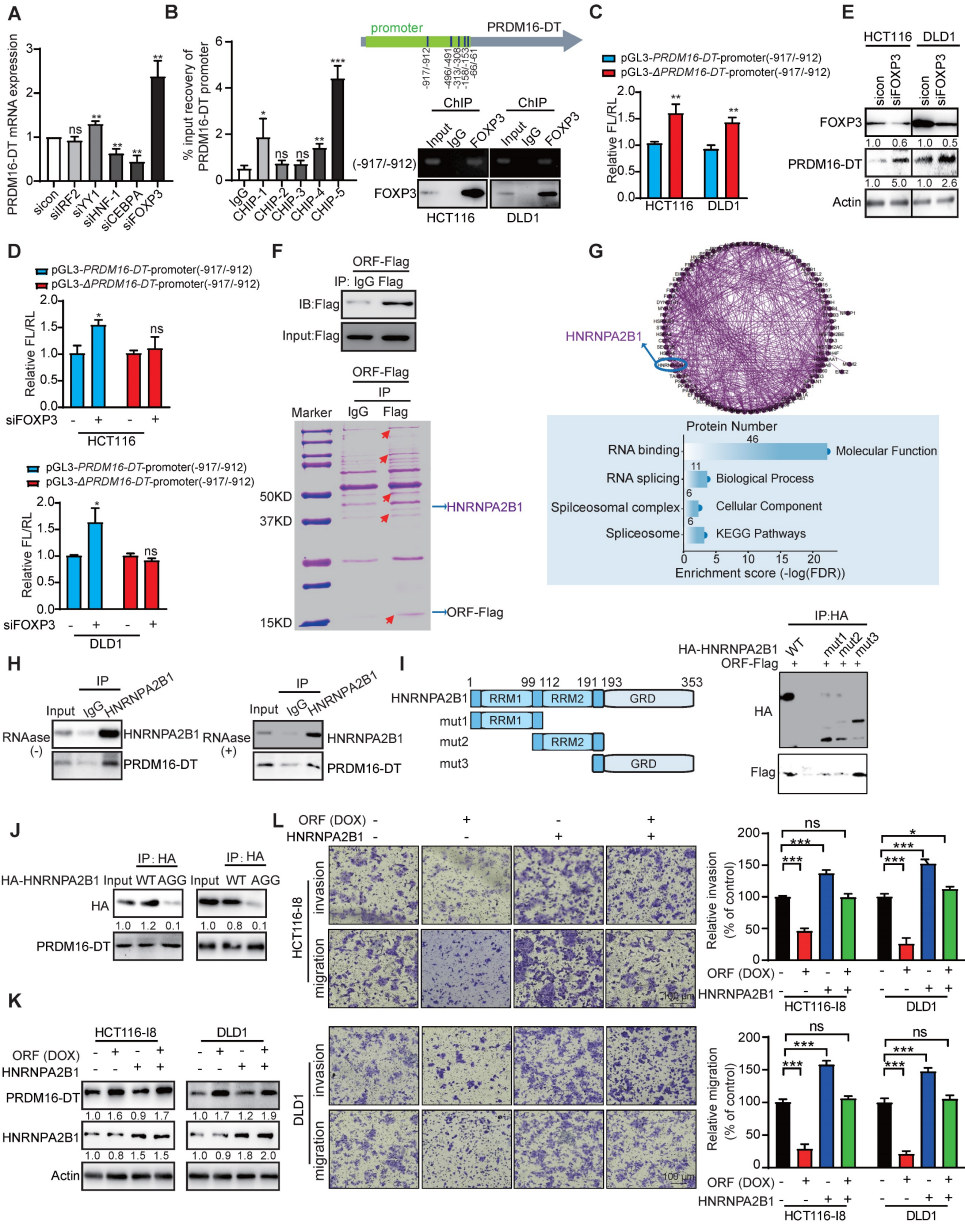
** PRDM16-DT, transcriptionally regulated by FOXP3, suppresses cancer metastasis by interacting with HNRNPA2B1. (A)** Predicted IRF2, YY1, HNF-1, CEBPA, and FOXP3-binding motifs located at the promoter of PRDM16-DT. CRC cells were treated with siRNAs (100 nM) of IRF2, YY1, HNF-1, CEBPA, and FOXP3 for 48 h. qRT-PCR assay shows marked upregulation of PRDM16-DT expression by siFOXP3. n = 3/experiments. **(B)** ChIP assay to determine FOXP3 binding with -917/-912 region of the PRDM16-DT promoter. n = 3/experiments. **(C)** Increased transcriptional activity of construct with -917/-912 deleted (Δ*PRDM16-DT*) in CRC cells. FL: Firefly luciferase; RL: Renilla luciferase. n = 3/experiments. **(D)** Increased transcriptional activity of the PRDM16-DT reporter construct with the intact region (-917/-912) following FOXP3 knockdown. n = 3/experiments. **(E)** Upregulation of PRDM16-DT in CRC cells following FOXP3 knockdown. n = 3/experiments. **(F)** PRDM16-DT immuno-precipitates in CRC cells on a gel. **(G)** Enrichment of RNA splicing in the proteins interacting with PRDM16-DT. n = 3/experiments. **(H)** Co-IP assay with anti-HNRNPA2B1 antibodies showing the endogenous interaction of HNRNPA2B1 with PRDM16-DT, even in the presence of RNAase treatment (30 mg/mL RNAase for 30 min). IgG was used as a control. n = 3/experiments. **(I)** An HA tag was added to the truncated fragments of HNRNPA2B1, and Co-IP assay with anti-HA antibodies showing reduced binding between PRDM16-DT and HNRNPA2B1 upon GRD domain deletion. IgG was used as a control. n = 3/experiments. **(J)** Co-IP assay to analyze the binding of PRDM16-DT with wild-type HNRNPA2B1 and its AGG mutant in CRC cells. n = 3/experiments. **(K-L)** HNRNPA2B1 overexpression in CRC cells treated with DOX (1 mg/mL, 48 h) **(K)** Significant abrogation of the inhibitory effect of PRDM16-DT on CRC cells by HNRNPA2B1 **(L)**. n = 3/experiments. Scale bar, 100 μm. Bars, SD; **, P < 0.01; ***, P < 0.001; ns, no significant difference.

Since tumor tissues expressed lower levels of LINC00982 and PRDM16-DT than normal tissues, we investigated whether transcriptional regulation was involved in the downregulation of PRDM16-DT in CRC. To gain insights into the transcriptional mechanisms involved, the PRDM16-DT promoter was predicted by promoter 2.0. Subsequently, we analyzed the predicted promoter of PRDM16-DT for transcription factor binding sites, and detected IRF2, YY1, HNF-1, CEBPA, and FOXP3-binding motifs located in the PRDM16-DT promoter region. Among these transcription factors, since FOXP3 knockdown most markedly upregulated the PRDM16-DT expression (Figure 3A), we speculated that FOXP3 might act as a transcription repressor of PRDM16-DT. Furthermore, most tumor tissues showed high FOXP3 expression in various cancers (Figure S5A), and patients with high FOXP3 expression had shorter survival than those with low FOXP3 expression (Figure S5B).

We performed ChIP assays using anti-FOXP3 or IgG control antibodies and a primer set (ChIP-1, ChIP-2, ChIP-3, ChIP-4, ChIP-5) for the PRDM16-DT predicted promoter region -1000 to -1 bp. Our results demonstrated FOXP3 binding to the predicted -917/-912 PRDM16-DT promoter region (Figure 3B), which was required for transcriptional downregulation of PRDM16-DT as the transcriptional activity of the reporter construct was elevated when the region (-917/-912) was deleted (Δ*PRDM16-DT*) (Figure 3C). Moreover, the knockdown of FOXP3 selectively enhanced the transcriptional activity of the PRDM16-DT reporter (Figure 3D). The inhibitory effect of FOXP3 on PRDM16-DT expression was also confirmed at the protein level (Figure 3E), supporting the notion that FOXP3 transcriptionally represses PRDM16-DT. Additionally, PRDM16-DT levels were negatively linked with the FOXP3 expression levels in diverse cancer types (Figure S5C). These results demonstrated that FOXP3 is responsible for the downregulation of PRDM16-DT in cancer cells.

To explore the underlying mechanisms, IP-MS was employed to detect the PRDM16-DT interacting proteins (Figure 3F). Analysis of the proteins identified from mass spectrometry indicated that PRDM16-DT may regulate RNA splicing by interacting with splicing regulators (Figure 3G). Among the candidates, HNRNPA2B1 was of interest as it was reported to regulate RNA splicing and act as the “reader” of m6A modification 17. CO-IP assay was used to further confirm that PRDM16-DT protein could interact with HNRNPA2B1, even in the presence of RNAase (Figure 3H), suggesting that the binding of PRDM16-DT to HNRNPA2B1 did not depend on RNA. HNRNPA2B1 had several recognizable substrate-binding domains, including two RNA recognition motifs (RRM1 and RRM2) and an RNA-binding RGG box (GRD). Next, we truncated HNRNPA2B1 with an HA tag and co-expressed the truncated fragments individually with PRDM16-DT to explore which domains were necessary for the binding. The results showed a diminished interaction between PRDM16-DT and HNRNPA2B1 upon deleting the GRD domain (mut1 and mut2) but not any other regions of HNRNPA2B1, suggesting that the GRD domain was required for the interaction of HNRNPA2B1 with PRDM16-DT (Figure 3I). It has been reported that interactions of RGG-motif proteins with other proteins can be charge-based. We thus mutated the arginine residues of three RGGs to alanine residues (AGG) in the GRD domain of HNRNPA2B1, constructed an HNRNPA2B1-AGG mutant plasmid, and overexpressed it and wild-type HNRNPA2B1 (WT) in CRC cells (Figure 3J). As displayed in Figure 3J, PRDM16-DT bound the wild-type HNRNPA2B1 but not the HNRNPA2B1-AGG mutant, suggesting that the arginine residues in the RGG motif were important for PRDM16-DT binding. Functionally, HNRNPA2B1 knockdown markedly reduced HCT116-I8 and DLD1 cell migration and invasion (Figure S6A-B).

We divided CRC patients (GSE20916, GSE17536) into two groups according to the HNRNPA2B1 status, and found that patients with HNRNPA2B1-high had high risk for poor prognosis (Figure S6C-D). Most tumor tissues had higher HNRNPA2B1 expression than adjacent normal tissues (Figure S6E). Kaplan-Meier survival analysis revealed significantly shorter survival in patients with high HNRNPA2B1 expression than those with low HNRNPA2B1 expression (P < 0.05, Figure S6F). The Receiver Operating Characteristic (ROC) curve illustrated that HNRNPA2B1 could be used as a diagnostic marker for CRC with an AUC value of 0.9289 (CPTAC, ID: PDC000116, Figure S6G). We further investigated whether HNRNPA2B1 mediated PRDM16-DT function by transfecting the HNRNPA2B1-overexpressing plasmid and vector control into CRC cells. Subsequently, cell migration, invasion, and oxaliplatin resistance were compared following DOX treatment (Figure 3K). The results indicated that HNRNPA2B1 overexpression significantly abolished the inhibitory effect of PRDM16-DT on CRC cell migration, invasion, and oxaliplatin resistance (Figure 3L, Figure S6H), suggesting that PRDM16-DT protein interacts with HNRNPA2B1 to suppress cancer metastasis and resistance to oxaliplatin.

### FOXP3/PRDM16-DT/HNRNPA2B1 regulatory axis serves as a favorable biomarker in CRC metastasis and chemoresistance

To study the correlation between FOXP3, HNRNPA2B1, and PRDM16-DT and CRC metastasis, we detected the expression of FOXP3, HNRNPA2B1, and PRDM16-DT in a tissue microarray (TMA) containing 30 cases of CRC T/M/N stages by multiplex immunohistochemical (mIHC) analysis. We found that most primary tissues (T1 & 2, T3 & 4) had higher PRDM16-DT and lower FOXP3 and HNRNPA2B1 expression than the metastatic tissues (M) (Figure S7A). On the contrary, low PRDM16-DT and high FOXP3 and HNRNPA2B1 expression were positively correlated with advanced stage (T1 & 2 vs T3 & 4) and metastasis (T1 & 2 vs M, T3 & 4 vs M) in cancer patients (Figure S7B), suggesting that FOXP3/PRDM16-DT/HNRNPA2B1 regulatory axis is associated with CRC distal metastasis.

The correlation between FOXP3/PRDM16-DT/HNRNPA2B1 regulatory axis and chemoresistance was explored by mIHC analysis using a TMA containing 10 cases each of oxaliplatin-resistant and oxaliplatin-sensitive CRC tissues. The data showed that the majority of the oxaliplatin-sensitive CRC tissues had a higher PRDM16-DT and lower FOXP3 and HNRNPA2B1 expression than the oxaliplatin-resistant CRC tissues (Figure S7C-D), corroborating the correlation between the FOXP3/PRDM16-DT/HNRNPA2B1 regulatory axis and chemoresistance. Collectively, these results suggest that the FOXP3/PRDM16-DT/HNRNPA2B1 regulatory axis is a favorable biomarker in CRC metastasis and chemoresistance.

### PRDM16-DT promotes *CHEK2* exon 9 inclusion by interacting with HNRNPA2B1

**Figure 4:**
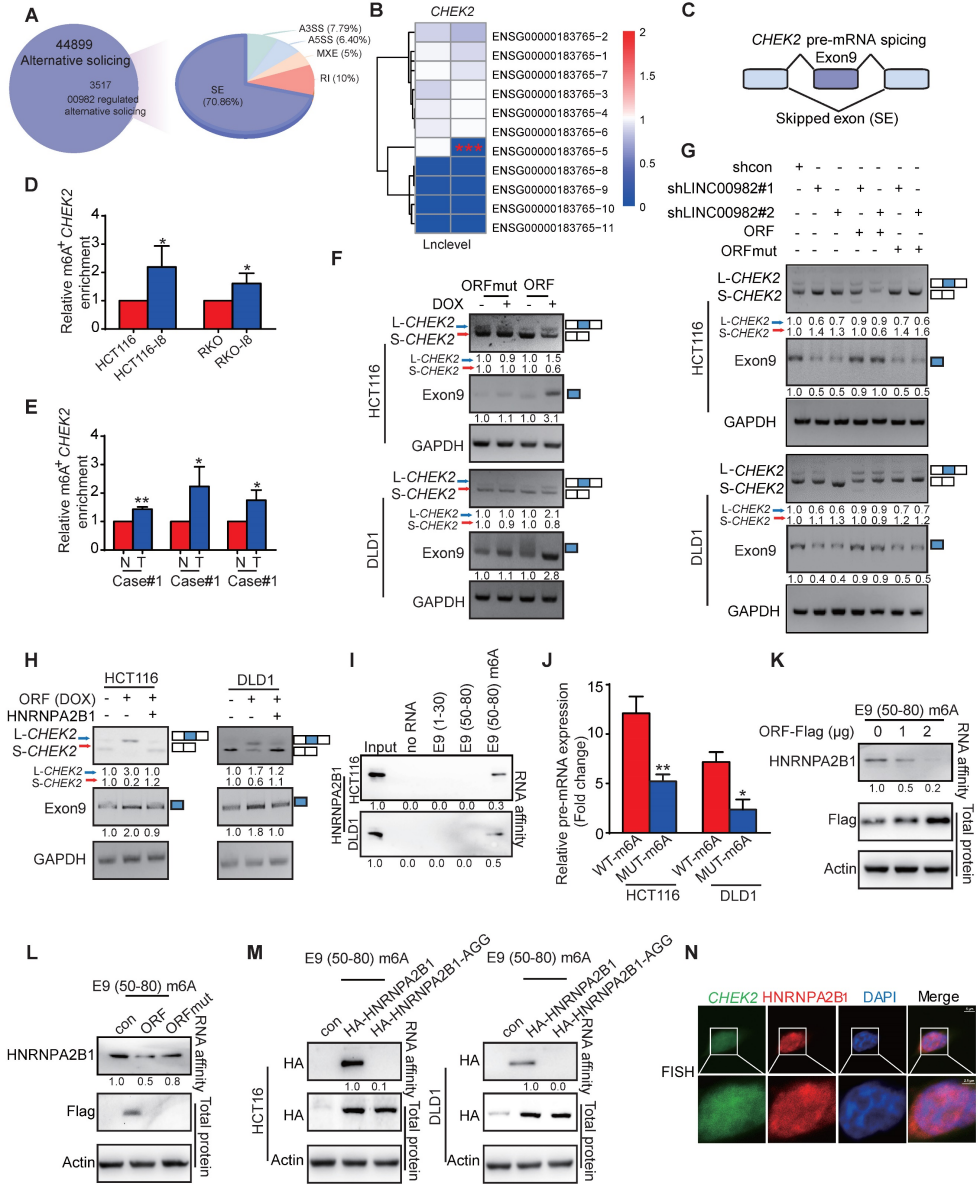
** PRDM16-DT promotes the formation of long *CHEK2* (L-*CHEK2*). (A)** PRDM16-DT-mediated AS events of mRNAs analyzed by RNA-seq. (B-C) RNA-seq showing the inclusion of *CHEK2* exon 9 (E9) promoted by PRDM16-DT. (D-E) MeRIP-qPCR analysis to validate the higher m6A modification of *CHEK2* in I8 cells **(D)** and tumor tissues **(E)** than parental cells and normal tissues. n = 3/experiments. **(F)** RT-PCR assay showing the inclusion of exon 9 of *CHEK2* promoted by PRDM16-DT. n = 3/experiments. **(G)** LINC00982 knockdown cells were transfected with the indicated plasmid, and the RT-PCR assay showed that knockdown of LINC00982 suppressed the inclusion of exon 9 of *CHEK2*, restored expression with ORF but not ORFmut reversed the effect. n = 3/experiments. **(H)** CRC cells treated with DOX (1 mg/mL) for 48 hours were transfected with HNRNPA2B1 overexpression plasmid. RT-PCR assay showing that HNRNPA2B1 determined the PRDM16-DT-regulated splicing of *CHEK2.* **(I)** RNA pulldown assay showing a strong interaction between HNRNPA2B1 and E9 (50-80) labeled with m6A modification. n = 3/experiments. **(J)** RIP assay confirming the weaker binding of HNRNPA2B1 and E9 with mutation on its m6A-binding site. n = 3/experiments. **(K)** Cells were transfected with ORF plasmid (0 μg, 1 μg, 2 μg). RNA pulldown assay shows decreased binding of HNRNPA2B1 to *CHEK2* in an ORF dose-dependent manner. n = 3/experiments. **(L)** ORF and ORFmut vectors were transfected into CRC cells. RNA pulldown assay shows markedly reduced binding of HNRNPA2B1 to *CHEK2* exon 9 by ORF but not ORFmut. n = 3/experiments. **(L)** Lack of the AGG mutant of HNRNPA2B1 binding to *CHEK2*. n = 3/experiments. **(N)** FISH assay showing HNRNPA2B1 co-localization with *CHEK2*. n = 3/experiments. Scale bar, 5 μm; 2.5 μm. Bars, SD; **, P < 0.01; ***, P < 0.001.

PRDM16-DT-mediated AS events of mRNAs were analyzed by RNA-seq. Figure 4A shows that PRDM16-DT overexpression regulated 3517 splicing events, including A3SS, A5SS, MXE, RI, and SE. We focused on PRDM16-DT-related pre-mRNA splicing and skipped exon (SE) because of its highest percentage among various events (Figure 4A). It has been reported that HNRNPA2B1 regulated the splicing events of pre-mRNA subjected to N6-methyladenosine (m6A) modification by binding to the RGAC sequences 17. Among the candidates, exon 9 (E9) of *CHEK2*, containing the HNRNPA2B1-binding motif sequence GGACT (Figure 4B-C), was further explored. The MeRIP-qPCR results confirmed the presence of m6A modification in *CHEK2*, with a significant increase in m6A level in I8 cells compared to parental CRC cells (Figure 4D). Furthermore, tumor tissues showed higher m6A levels of *CHEK2* than normal tissues (Figure 4E), suggesting that the m6A level may be related to PRDM16-DT function. RT-PCR assay showed that PRDM16-DT promoted *CHEK2* exon 9 inclusion, leading to the formation of a long *CHEK2* (L-*CHEK2*) splicing variant and the simultaneous suppression of short *CHEK2* (S-*CHEK2*) splicing variant (Figure 4F). In contrast, LINC00982 knockdown inhibited exon 9 inclusion in *CHEK2*, resulting in L-*CHEK2* suppression, and restored expression of ORF, significantly abrogating the suppression effect of LINC00982 silencing with no influence from the re-expression of ORFmut (Figure 4G). We also observed that HNRNPA2B1 overexpression decreased the PRDM16-DT-regulated splicing of *CHEK2* (Figure 4H). We explored the underlying mechanism by which PRDM16-DT promoted the inclusion of exon 9 in *CHEK2* by performing Western blotting to detect HNRNPA2B1 and PRDM16-DT expression. The results showed that PRDM16-DT did not change the expression level of HNRNPA2B1 (Figure 3F). Therefore, we speculated that PRDM16-DT might mediate the binding of HNRNPA2B1 to *CHEK2*. An RNA pulldown assay using biotin-labeled RNA Corresponding to E9 (1-30) and (50-80) without m6A modification showed no binding (Figure 4I). Since the m6A motif sequence is in *CHEK2* E9 (50-80), we further investigated whether m6A modification was necessary for the binding of HNRNPA2B1 and *CHEK2*. As displayed in Figure 4I, a strong interaction was observed between HNRNPA2B1 and E9 (50-80) labeled with biotin and m6A modification, suggesting that the *CHEK2* exon 9 with m6A modification was essential for HNRNPA2B1 binding. Consistently, RIP assay also confirmed the weaker binding of HNRNPA2B1 and E9 in which mutation was made on its m6A-binding site, implying that the m6A sequence motif GGACT plays a significant role in the interaction between HNRNPA2B1 and exon 9 of *CHEK2* (Figure 4J).

We investigated the influence of PRDM16-DT on the binding of HNRNPA2B1 to *CHEK2* and found that the binding reduced in a PRDM16-DT-dose-dependent manner (Figure 4K). Meanwhile, PRDM16-DT (ORF) but not LINC00982 (ORFmut) markedly reduced HNRNPA2B1 binding to *CHEK2* exon 9 (Figure 4L). Also, the HNRNPA2B1 AGG mutant did not bind to the *CHEK2* mRNA sequence (Figure 4M), indicating that the arginine residue in the RGG motif of HNRNPA2B1 was required of the binding. In addition, the FISH assay confirmed that HNRNPA2B1 was co-localized with *CHEK2* (Figure 4N). Collectively, these data demonstrated that PRDM16-DT protein blocks the interaction of HNRNPA2B1 with *CHEK2* by competitively binding to the RGG motif of HNRNPA2B1.

### L-*CHEK2*, not S-*CHEK2*, suppresses CRC metastasis and reverses drug resistance

**Figure 5:**
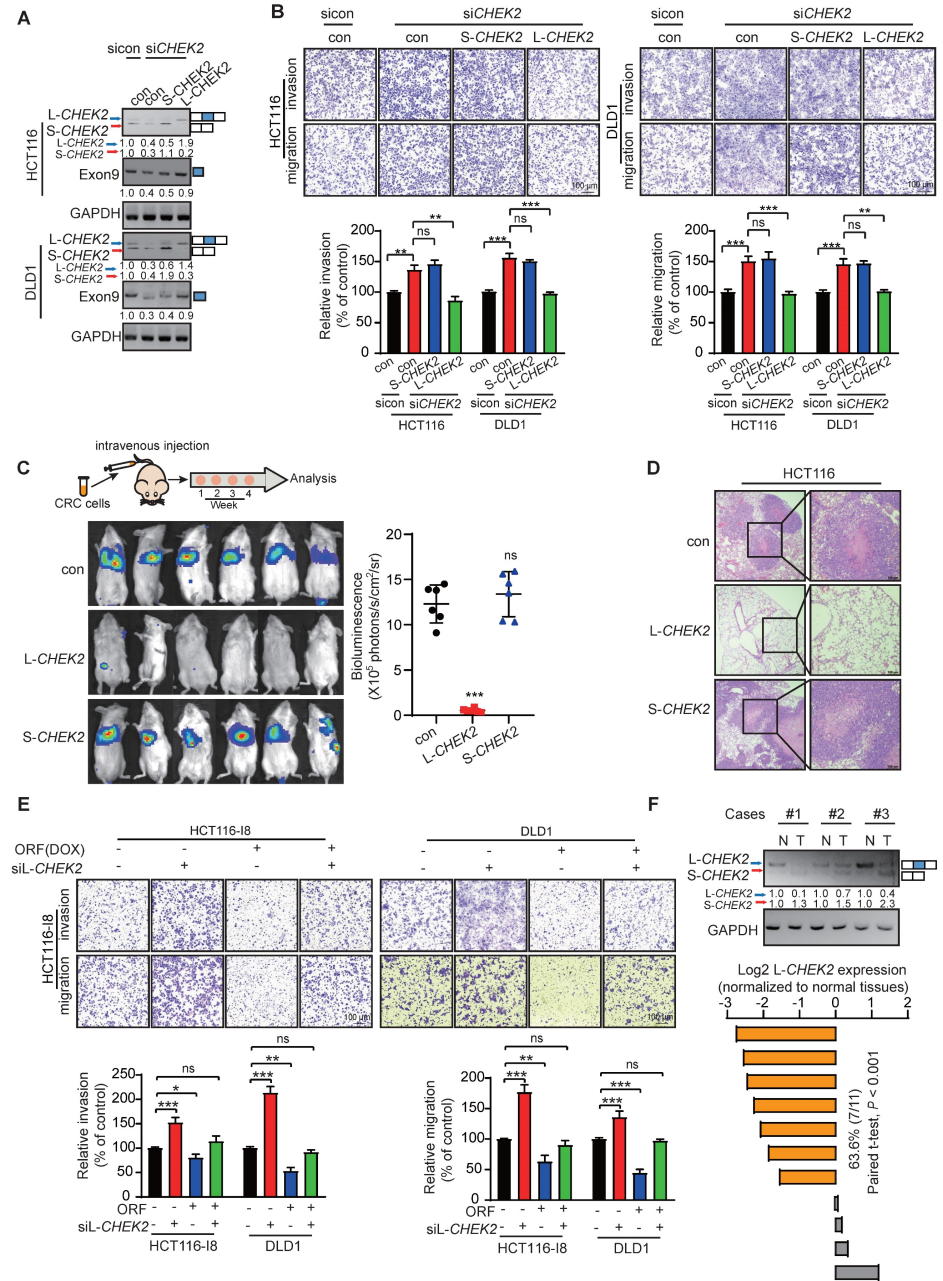
** L-*CHEK2*, not S-*CHEK2*, suppresses metastasis of CRC. (A-B)** siRNA (100 nM) of *CHEK2* with L-*CHEK2* or S-*CHEK2* vector were transfected in CRC cells **(A)**; increased cell migration and invasion by *CHEK2* silencing, which can be repressed to the control level upon re-expression with L-*CHEK2* **(B)**. n = 3/experiments. Scale bar, 100 μm. **(C-D)** CRC cells with stable overexpression of L-*CHEK2* or S-*CHEK2* were intravenously injected into mice *via* the tail vein, after 4 weeks, the reduced metastatic potential in mice by L-*CHEK2* was analyzed by bioluminescence imaging (n = 6 mice/group) **(C)**, and H&E staining (n = 3 mice/group) **(D)**, Scale bar, 100 μm. **(E)** Knockdown of L-*CHEK2* in CRC cells treated with DOX (1 mg/mL, 48 h) showing efficient restoration of cell metastasis to control levels reduced by ORF overexpression. n = 3/experiments. Scale bar, 100 μm. **(F)** QRT-PCR assay showing decreased L-*CHEK2* mRNA levels in tumor tissues compared to normal tissues. Bars, SD; **, P < 0.01; ***, P < 0.001; ns, no significant difference.

The biological functions of L-*CHEK2* and S-*CHEK2* in CRC were assessed by performing metastasis and cell viability assays. The results showed that *CHEK2* silencing increased cell migration, invasion, and resistance to oxaliplatin, which could be reverted to control levels upon re-expression of L-*CHEK2* but not S-*CHEK2* (Figure 5A-B, Figure S8A-B), indicating the crucial role of exon 9 in the biological function of *CHEK2*. Next, CRC cells with stable overexpression of L-*CHEK2* or S-*CHEK2* were intravenously injected into mice *via* the tail vein, and signals were monitored by bioluminescence imaging. Notably, L-*CHEK2* markedly suppressed lung metastasis, as confirmed by H&E staining (Figure 5C-E). In contrast, S-*CHEK2* did not affect the metastatic ability of CRC cells as compared with the control group (Figure 5C-D). Also, L-*CHEK2* silencing efficiently restored cell migration and invasion and oxaliplatin resistance decreased by overexpressing PRDM16-DT (Figure 5E, Figure S8C). We determined the clinical significance of L-*CHEK2* in CRC by analyzing its expression in tumor samples. The qRT-PCR assay showed decreased L-*CHEK2* mRNA levels in tumor tissues compared to their matched adjacent normal tissues (Figure 5F). Furthermore, L-*CHEK2* expression was downregulated in primary tumors (T3 & 4) and further decreased in metastatic tumors as compared with T1 & 2 primary tumors, indicating its association with the advanced tumor stage and metastasis (Figure S8D). The association of L-*CHEK2* with chemoresistance was also evident, showing the downregulation of L-*CHEK2* expression in oxaliplatin-resistant CRC tissues compared to oxaliplatin-sensitive CRC tissues (Figure S8E). Given the important role of *CHEK2* in DNA repair, camptothecin (CPT) was used to induce DNA damage, and single-cell gel electrophoresis was performed. As shown in Figure S8F, low levels of DNA damage were observed in L-*CHEK2*- and S-*CHEK2*-overexpressing cells, suggesting that altered *CHEK2* splicing did not affect DNA repair. These data indicated that PRDM16-DT suppresses tumor formation/progression mainly by inhibiting *CHEK2* splicing.

### PRDM16-DT promotes E-cadherin secretion to inhibit fibroblast activation in a paracrine manner

**Figure 6:**
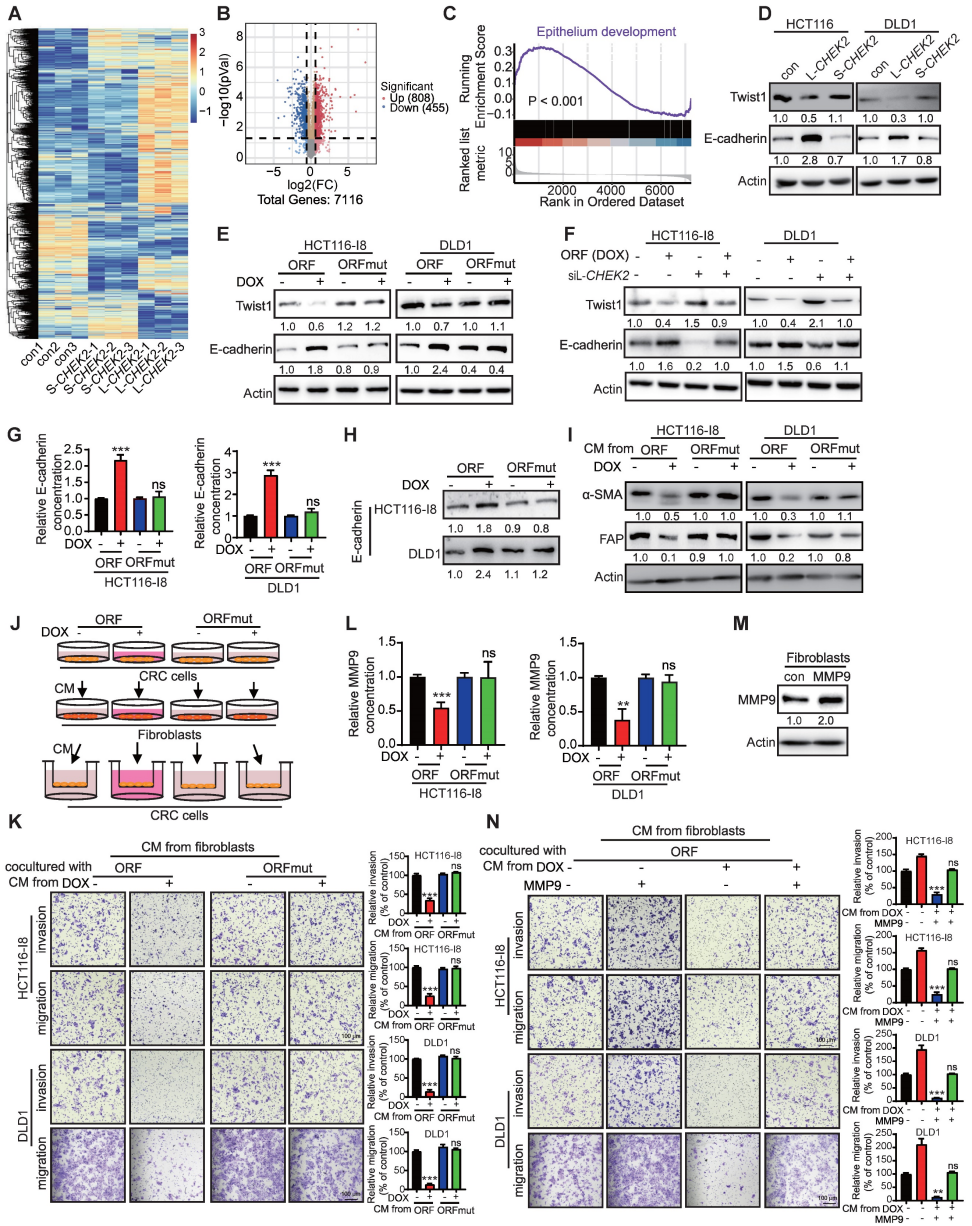
** PRDM16-DT promotes E-cadherin secretion to inhibit fibroblast activation in a paracrine manner. (A)** Heatmap of gene profiles in L-*CHEK2* and S-*CHEK2*-overexpressing CRC cells. **(B)** Volcano plot of DEPs regulated by L-*CHEK2*. **(C)** GSEA analysis showing EMT enrichment in DEGs of L-*CHEK2*-overexpressing cells. **(D)** Expression of EMT markers (Twist, E-cadherin) in L-*CHEK2*-overexpressing cells compared with S-*CHEK2*-overexpressing and control cells. n = 3/experiments. **(E)** Decreased Twist expression and increased E-cadherin expression in ORF-overexpressing CRC cells, with no expression change in ORFmut cells. n = 3/experiments. **(F)** Reversal of effects of EMT markers in ORF-overexpressing cells by L-*CHEK2* silencing. n = 3/experiments. **(G-H)** Increased E-cadherin secretion in the conditioned medium (CM) from ORF-overexpressing CRC cells compared to control cells by ELISA **(G)** and Western blotting **(H)**. n = 3/experiments. **(I)** Decreased expression of α-SMA and FAP in the CM from ORF-overexpressing CRC cells by Western blotting. n = 3/experiments. **(J)** Schematic diagram for coculture system. **(K)** Invasive ability of CRC cells treated with CM from different fibroblasts as indicated by the metastasis assay. n = 3/experiments. Scale bar, 100 μm. **(L)** Decreased MMP9 secretion in fibroblasts cocultured with CM from ORF-overexpressing CRC cells compared to control cells by ELISA. n = 3/experiments. **(L)** MMP9 overexpression in fibroblasts. n = 3/experiments. **(N)** Metastasis assay showing MMP9 overexpression in fibroblasts and reversal of the inhibitory effect of fibroblasts cultured with CM from ORF-overexpressing CRC cells on the metastasis ability of CRC cells as indicated. n = 3/experiments. Scale bar, 100 μm. Bars, SD; **, P < 0.01; ***, P < 0.001.

We performed DIA-mass spectrometry on CRC cells to explore the downstream mechanisms by which L-*CHEK2* inhibits cancer metastasis. Key signaling pathways mediated by L-*CHEK2* overexpression were examined in CRC cells and S-*CHEK2*-overexpressing cells compared with control cells. Clustering analysis showed significantly different patterns of protein profiles in L-*CHEK2*- and S-*CHEK2*-overexpressing cells (Figure 6A). A total of 1263 DEPs were identified, including 455 down-regulated and 808 upregulated (fold-change ≥ 1.5, P ≤ 0.05, Table S3, Figure 6B). KEGG analysis on the DEPs was performed to identify signaling pathways affected by L-*CHEK2*, indicating that EMT might play a significant role in the anti-metastatic activity of L-*CHEK2* (Figure 6C). Western blotting analysis confirmed L-*CHEK2*-induced downregulation of the mesenchymal marker Twist and upregulation of the epithelial marker E-cadherin (Figure 6D). Decreased expression of Twist and increased expression of E-cadherin in ORF-overexpressing CRC cells was also observed, but there was no change in ORFmut-overexpressing cells (Figure 6E). Furthermore, as shown in Figure 6F, the effects of ORF-overexpression on EMT markers were eliminated by L-*CHEK2* silencing. These data proved that PRDM16-DT regulates EMT through L-*CHEK2*. We investigated the E-cadherin secretion level in ORF-overexpressing CRC cells since it is a key factor for cell-cell crosstalk 19, 20. The enzyme-linked immunosorbent assay (ELISA) and Western blotting revealed that E-cadherin secretion increased in the conditioned medium (CM) of ORF-overexpressing (DOX+) CRC cells compared to the CM from control cells (DOX-) and ORFmut-overexpressing CRC cells (Figure 6G-H). Moreover, the migration and invasion assays showed that CM from ORF-overexpressing CRC cells not only reduced the fibroblast metastasis (Figure S9A-B), but also decreased the expressions of fibroblast markers a-smooth muscle actin (α-SMA) and fibroblast activation protein (FAP) (Figure 6I). No expression changes of these factors were observed in the CM from ORFmut-overexpressing CRC cells (Figure S9A-B, Figure 6I), indicating that PRDM16-DT inhibited fibroblast activation. Further, this effect was attenuated by treatment with anti-E-cadherin-neutralizing antibody (Figure S9C), suggesting that E-cadherin is essential for the inhibitory effect of PRDM16-DT on fibroblast activation. A coculture system was used to examine whether fibroblasts inhibited by PRDM16-DT could affect CRC metastasis and resistance to oxaliplatin (Figure 6J). The results showed that the metastasis and oxaliplatin resistance of CRC were significantly reduced by coculturing with the CM from ORF-overexpressing CRC cells compared to the CM from control cells and ORFmut-overexpressing CRC cells (Figure 6K, Figure S9D), indicating that de-activation of fibroblasts could suppress CRC metastasis and oxaliplatin resistance. It has been reported that coculturing cancer cells with fibroblasts resulted in the induction of MMP9 expression associated with cancer development 19. The ELISA assay in Figure 6L revealed that MMP9 secretion in the CM from the coculture with ORF-overexpressing CRC cells decreased compared to the coculture with control cells and ORFmut-overexpressing CRC cells. Moreover, MMP9 overexpression could reverse the inhibitory effect of fibroblasts by ORF-overexpression in CRC cells (Figure 6M-N, Figure S9E). Collectively, these data indicated that PRDM16-DT regulates E-cadherin expression to mediate the crosstalk between CRC cells and fibroblasts.

### Cimicifugoside H-1 enhances sensitivity of CRC cells to oxaliplatin by directly targeting FOXP3

**Figure 7:**
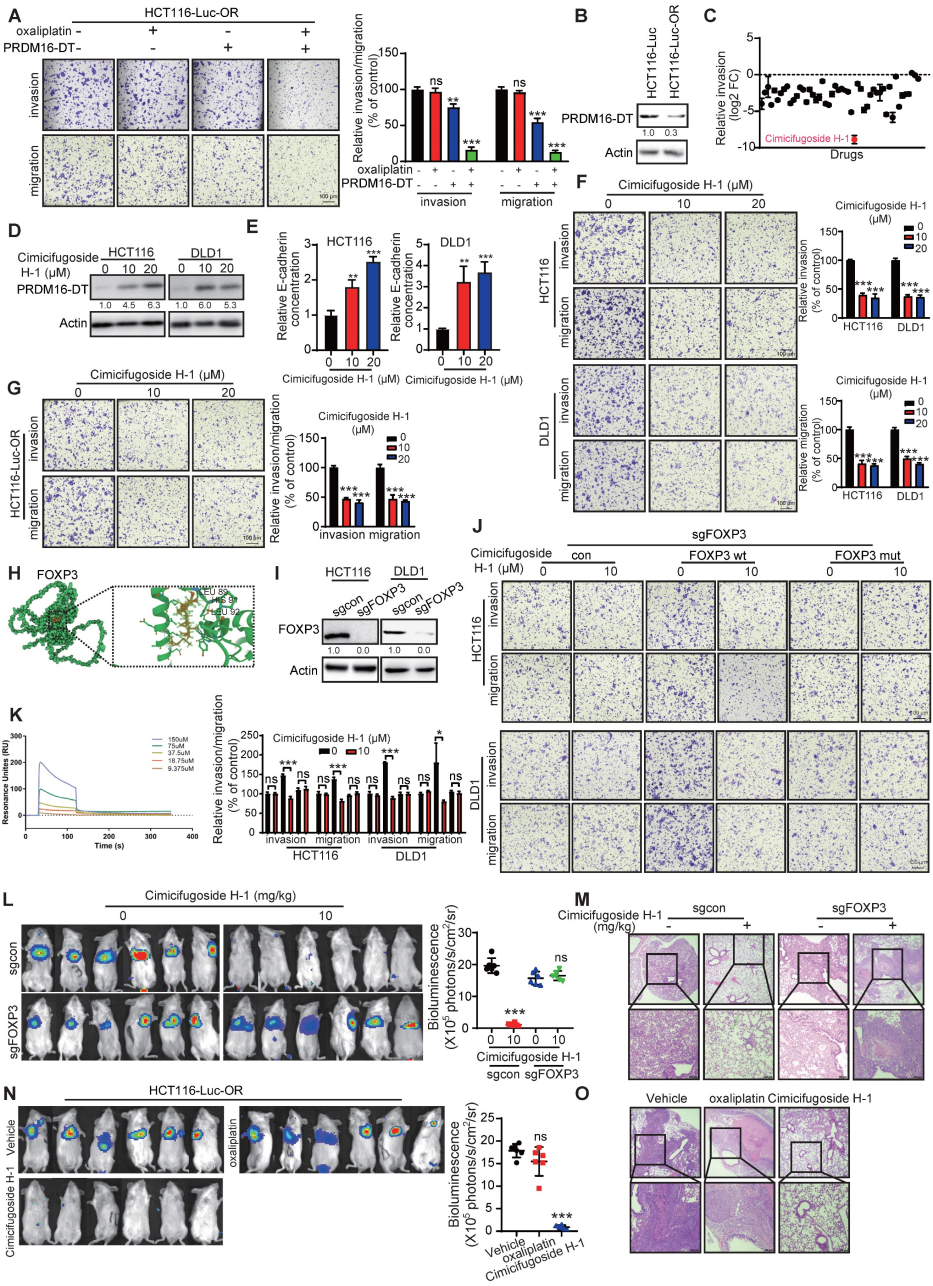
** Identification of cimicifugoside H-1 as a novel sensitizer for CRC oxaliplatin treatment. (A)** HCT116-Luc-OR cells transfected with PRDM16-DT-expressing plasmid (1 μg) or vector control were treated with oxaliplatin (20 μM) for 48 h. Metastasis assay showing significant and synergistic suppression of HCT116-Luc-OR metastasis by the combined treatment of PRDM16-DT overexpression with oxaliplatin. n = 3/experiments. Scale bar, 100 μm. **(B)** Low endogenous expression of PRDM16-DT in OR CRC tissue and cells. n = 3/experiments. **(C)** Identification of anticancer drugs from the library comprising 50 plant natural products (10 μM, 24h) using the invasion assay. Significant inhibitory effect of cimicifugoside H-1, among the 50 compounds tested on the invasion of HCT116 cells. n = 3/experiments. **(D)** Increased PRDM16-DT expression in CRC cells by cimicifugoside H-1 (10 μM, 20 μM, 24 h) as evidenced by Western blotting. n = 3/experiments. **(E)** Increased E-cadherin secretion by cimicifugoside H-1 (10 μM, 20 μM, 24 h). n = 3/experiments. (F-G) Migration assay showed that Significant inhibition of invasion and migration of CRC cells **(F)** and OR CRC cells **(G)** by cimicifugoside H-1 (10 μM, 20 μM, 24 h). n = 3/experiments. Scale bar, 100 μm. **(H)** Molecular docking to predict the binding sites of cimicifugoside H-1 with FOXP3 protein. The potential binding sites are labeled. **(I)** Establishment of FOXP3-deficient CRC cells. n = 3/experiments. **(J)** Overexpression of wild-type OR mutated FOXP3 in FOXP3-deficient CRC cells followed by metastasis assays to compare their sensitivity toward cimicifugoside H-1 treatment (10 μM, 24 h). n = 3/experiments. Scale bar, 100 μm. **(K)** Binding of FOXP3 with cimicifugoside H-1 (Kd=5.2×10^-8^) as determined by the Biacore assay. n = 3/experiments. FOXP3-deficient CRC and control cells were intravenously injected into mice *via* the tail vein. After one week, mice were divided into two subgroups for treatment with cimicifugoside H-1 (20 mg/kg) or the vehicle (0.5% CMC-Na). and lung metastasis was monitored. **(L)** No inhibition of lung metastasis by cimicifugoside H-1 in FOXP3-knockout cells. n = 6 mice/group. **(L)** H&E staining of the lungs. Mice were divided into three groups and treated by oral administration with vehicle (0.5% CMC-Na), cimicifugoside H-1 (20 mg/kg), or oxaliplatin (20 mg/kg). n = 3 mice/group. Scale bar, 100 μm. **(N)** HCT116-Luc-OR and control cells were intravenously injected into mice *via* the tail vein. After one week, mice were divided into three groups for treatment with cimicifugoside H-1 (20 mg/kg), oxaliplation (20 mg/kg) or the vehicle (0.5% CMC-Na), and lung metastasis was monitored. Superior suppressive effect of cimicifugoside H-1 compared to oxaliplatin on tumor metastasis. n = 6 mice/group. **(O)** H&E staining of the lungs. n = 3 mice/group. Scale bar, 100 μm. Bars, SD; **, P < 0.01; ***, P < 0.001.

As mentioned above, PRDM16-DT, transcriptionally regulated by FOXP3, suppressed cancer metastasis and chemoresistance. Given the vital role of cancer metastasis in the chemoresistance of CRC, we found PRDM16-DT overexpression significantly and synergistically suppressed the invasion and migration of OR CRC cells (Figure 7A). Correspondingly, a lower expression level of endogenous PRDM16-DT was observed in OR CRC cells compared to control cells (Figure 7B). Next, we screened new anticancer drugs targeting the FOXP3-PRDM16-DT axis to suppress CRC metastasis and reverse drug resistance. The CRC cell line, HCT116, was subjected to cell invasion analysis using a library comprising 50 plant natural products. Cimicifugoside H-1, a cyclolanostanol xyloside, was found to exert a significant inhibitory effect on cell invasion and was selected for further investigation (Figure 7C). Cimicifugoside H-1 increased PRDM16-DT expression and E-cadherin secretion, and significantly inhibited the invasion and migration of CRC and OR CRC cells (Figure 7D-G). Furthermore, WST-1 and colony-formation assays showed that cimicifugoside H-1 significantly inhibited the proliferation ability of OR CRC cells (Figure S10A-B), suggesting that cimicifugoside H-1 may be a novel strategy for alleviating oxaliplatin resistance.

To explore the underlying mechanism of cimicifugoside H-1, molecular docking predicted that FOXP3 may be the direct target of cimicifugoside H-1 with LEU89, HIS91, and LEU92 of FOXP3 being the most likely residues required for cimicifugoside H-1 binding (Figure 7H). For validation, we established FOXP3-deficient CRC cells (Figure 7I) and constructed a plasmid to express the FOXP3 mutant (with mutations of L89A, H91A, and L92A) and overexpressed the mutant and wild-type FOXP3 (FOXP3-wt) in FOXP3-deficient HCT116 and DLD1 cells. The sensitivity of FOXP3-deficient CRC cells to cimicifugoside H-1 was markedly restored when FOXP3-wt was re-expressed to a level comparable to that of parental cells (Figure 7J). Re-expression of FOXP3-mut did not have the rescue effect (Figure 7J), validating the critical role of L89, H91, and L92 of FOXP3 in the binding to cimicifugoside H-1. Also, Biacore assay showed that FOXP3 bound to cimicifugoside H-1 with a binding constant of Kd = 5.2 × 10^-8^ (Figure 7K).

We also Next, the role of FOXP3 in the anticancer properties of cimicifugoside H-1 was evaluated in animal models. FOXP3-deficient HCT116-Luc cells and control cells were intravenously injected into mice *via* the tail vein. Each group was further divided into two subgroups for treatment with cimicifugoside H-1 or the vehicle. Consistent with the *in vitro* data, bioluminescence showed that cimicifugoside H-1 did not inhibit lung metastasis of FOXP3-knockout cells (Figure 7L). Histological analysis of xenografts (Figure 7M) further confirmed that FOXP3 was required for the anticancer bioactivity of cimicifugoside H-1. There were no significant differences in serum alanine and aspartate aminotransferase levels between the treatment and control groups in mice (Figure S10C). Furthermore, the animal experiments showed that cimicifugoside H-1 markedly suppressed lung metastasis of OR CRC cells (HCT116-Luc-OR), and oxaliplatin did not exert any change on OR CRC cell metastasis (Figure 7N), as evidenced by H&E staining of the lungs (Figure 7O). Collectively, our data demonstrated that cimicifugoside H-1 enhances the sensitivity of CRC cells to oxaliplatin by directly targeting FOXP3 with no significant side effects.

## Discussion

Cancer metastasis and drug resistance are two major factors of cancer death in CRC. This study aimed to identify novel regulators involved in cancer metastasis and chemoresistance and explore useful anticancer compounds with favorable therapeutic efficacy. Recently, lncRNAs have emerged as critical players in tumorigenesis, cancer progression, metastasis, apoptosis, and drug resistance 21, 22, 23. Due to the remarkable advances in deep-sequencing technologies, there is increasing evidence showing that specific lncRNAs harbor concealed functional proteins/peptides involved in various diseases, including cancer. In 2019, we revealed the first hidden human proteome containing 314 lncRNA-encoded new proteins by applying full-length mRNA sequencing, ribosome profiling, and shotgun proteomics in several stable cancer cell lines 11. Subsequently, a few lncRNA-encoded small proteins/peptides have been shown to play functional roles in tumor development and progression 24, 25. We hypothesized that some of these lncRNA-derived proteins may be crucial regulatory factors in cancer metastasis and initiated a systematic functional characterization. We previously reported that SMIM26, encoded by LINC00493, exerts anti-metastatic activity by inhibiting AGK-mediated AKT phosphorylation in renal cell carcinoma 26.

In this study, we established another metastatic model with highly invasive CRC cells and carried out functional screening on the model subjected to a CRISPR/Cas9 library targeting 314 lncRNAs.

LINC00982 was selected for further functional characterization because of the significant increase of LINC00982-targeted sgRNA in sgRNA-transduced cells (Figure 2C). It has been reported that LINC00982, as an lncRNA, plays important roles in regulating cancer phenotypes, including proliferation, apoptosis, metastasis, migration, and invasion 27, 28, 29, 30. In the present study, we experimentally verified, for the first time, that LINC00982 encodes a functional protein, PRDM16-DT to regulate cancer metastasis and chemoresistance. LINC00982, a divergent transcript of Prdm16, is located about 0.5 kb telomeric in the 5' untranslated region of Prdm16, and the novel protein was encoded by a 444-nucleotide ORF in LINC00982, we thus named this protein PRDM16-DT. However, there is no sequence homology between the nucleotides of the CDS region of Prdm16 and PRDM16-DT. Furthermore, there is no homology between the amino acid sequences of the two proteins.

Our cellular and molecular experimental evaluations confirmed that LINC00982 protein PRDM16-DT, not lncRNA, functioned as a tumor suppressor in CRC metastasis and chemoresistance (Figures 2 & 3, Figure S4). PRDM16-DT expression was transcriptionally regulated by FOXP3, showing its decreased level in CRC tumors and further down-regulation in metastatic tissues (Figures 2&4, Figure S6). It has been reported that FOXP3 can act as a transcription factor by forming dynamic supramolecular complexes with many transcription factors or enzymes, thereby performing transcriptional regulatory functions 31, 32. Here we revealed that FOXP3 binds to the -917/-912 region of the PRDM16-DT promoter to suppress the PRDM16-DT transcription as observed in ChIP and Luciferase reporter assays (Figure 3). Also, low PRDM16-DT expression was associated with poor prognosis of CRC patients, implicating the potential of PRDM16-DT as a biomarker in CRC diagnosis and prognosis.

We employed MS combined with CO-IP and found that PRDM16-DT exerted its biological function by interacting with HNRNPA2B1, which plays an important role in regulating transcription, transport, and AS events of pre-mRNA, contributing to cancer progression. HNRNPA2B1 is usually overexpressed in cancers and affects various malignant phenotypes 33, 34, 35, 36, regulating the AS of BIRC5 to induce BIRC5-202 expression and promoting GC cell growth and metastasis 37. More importantly, HNRNPA2B1 is known as a “reader” of m6A modification 17. Our study further demonstrated that HNRNPA2B1 regulated m6A-dependent anti-metastatic AS of *CHEK2* in CRC. By interacting with HNRNPA2B1 with the RGD motif, PRDM16-DT competitively inhibited the HNRNPA2B1 binding to *CHEK2* exon 9, resulting in L-*CHEK2* upregulation and suppressing CRC metastasis.

Tumor microenvironment (TME) is a master regulator of drug resistance and cancer metastasis. Fibroblasts, one of the most abundant cell types in TME, can be activated by appropriate factors to become cancer-associated fibroblasts (CFAs), which play a critical role in tumor metastasis by secreting cytokines. Previous studies have documented a crosstalk of fibroblasts with cancer cells 19, 38. For example, slow-cycling cancer cells (SCC) with chemotherapy treatment enhanced the secretion of various cytokines to recruit fibroblasts, subsequently activated to trigger cancer progression 38. However, the underlying mechanism of the crosstalk between fibroblasts and cancer cells remains to be elucidated. Herein, we revealed that PRDM16-DT increased E-cadherin secretion in cancer cells to reduce fibroblast activation and decrease MMP9 release, suppressing CRC metastasis. This observation illustrated the specific nature of the crosstalk between fibroblasts and cancer cells mediated by PRDM16-DT.

Plant natural products have a historically proven important value in the novel anticancer drug development. Given the significant role of PRDM16-DT in regulating CRC metastasis and chemoresistance, we identified a natural product, cimicifugoside H-1, which effectively suppressed CRC invasion by targeting FOXP3, the upstream regulator of PRDM16-DT. We further elucidated that cimicifugoside H-1 directly interacted with FOXP3 by binding to its LEU89, HIS91 and LEU92 residues. Cimicifugoside H-1 displayed a profound anticancer effect on cancer metastasis and drug resistance of CRC to oxaliplatin.

## Conclusions

**Figure 8:**
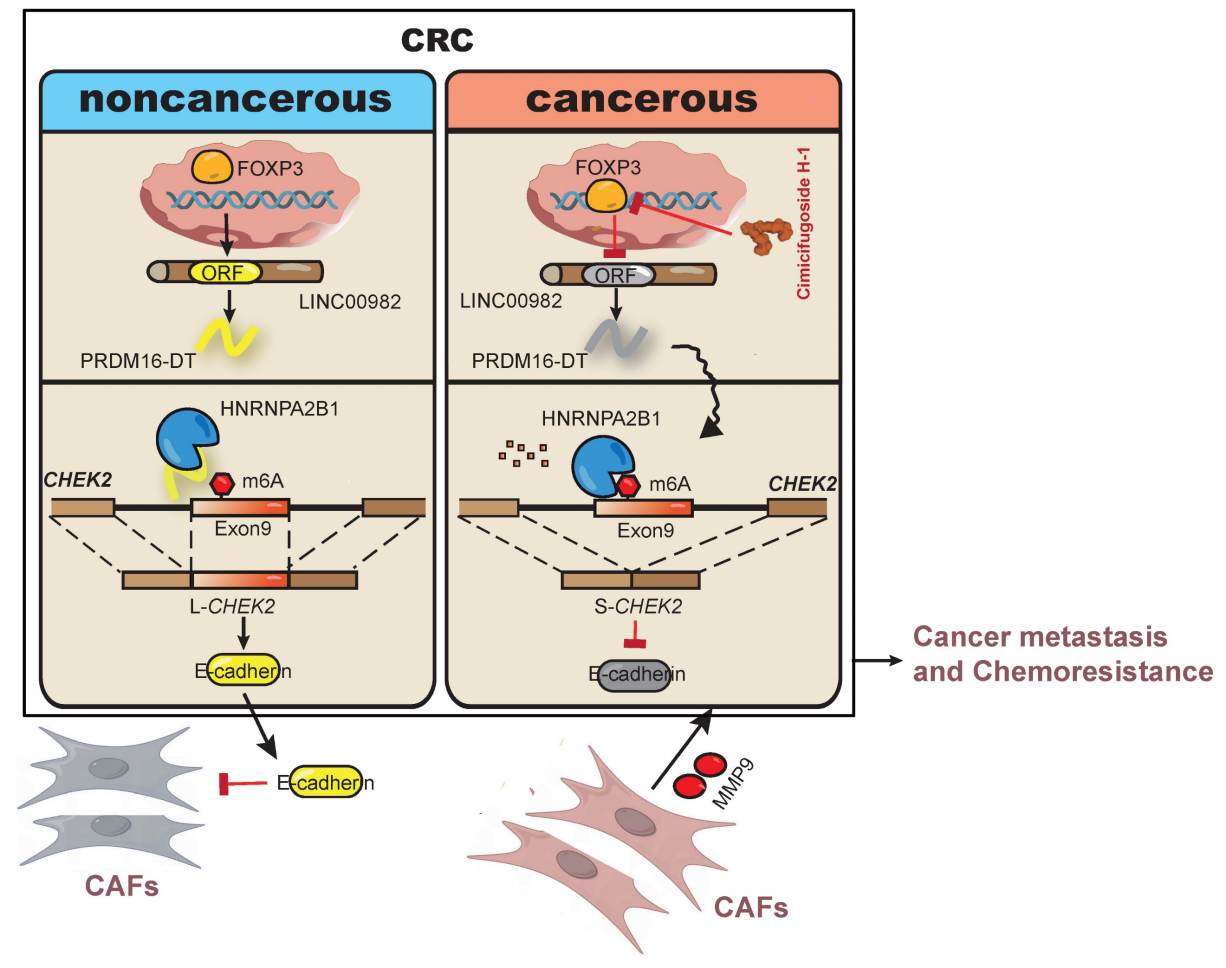
Schematic diagram summarizing cancer metastasis and chemoresistance suppression by PRDM16-DT.

As illustrated in Figure 8, our study discovered that PRDM16-DT encoded by LINC00982 functions as a CRC metastasis suppressor and a sensitizer of oxaliplatin resistance. PRDM16-DT could block HNRNPA2B1-mediated *CHEK2* splicing, inducing the subsequent formation of L-*CHEK2* and increasing the secretion of E-cadherin, thereby inhibiting fibroblast activation. Inactivation of fibroblasts by PRDM16-DT reduced the release of MMP9, thus suppressing CRC metastasis and oxaliplatin resistance. By targeting FOXP3, the transcription factor of PRDM16-DT, natural compound cimicifugoside H-1 promoted PRDM16-DT expression to inhibit CRC metastasis and reverse oxaliplatin resistance, implicating its potential as an efficient strategy for CRC therapy.

## Materials and Methods

### Cell culture

Human CRC cell lines HCT116, DLD1, HT29, and RKO were obtained from the American Type Culture Collection (Rockville, MD, USA). HCT116-I8 and RKO-I8 cell sublines with highly metastatic abilities were established in our previous study 18. NCM460, the normal colonic epithelial cell line, was purchased from INCELL Corporation (San Antonio, TX, USA). Cells were maintained in RPMI 1640 (Life Technologies, Gaithersburg, MD, USA) containing 10% fetal bovine serum (FBS) (Life Technologies) at 37 °C in 5% CO_2_ atmosphere. Oxaliplatin and cimicifugoside H-1 were obtained from Topscience (Shanghai, China) and dissolved in Dimethyl sulfoxide.

### CRISPR/Cas9 screening

CRISPR knockout pooled library targeting 314 lncRNAs was designed and constructed by Fubio Biological Technology Co., Ltd. (Shanghai, China). As previously described 39, cells were transduced and treated with puromycin (5 μg/mL). After one week, the input DNA of CRC cells (7×10^7^) was isolated using the DNA extraction and purification kit (Qiagen, Hilden, Germany). After three rounds of screening, highly invasive cells (7×10^7^) were harvested to obtain genomic DNA, as described previously 18. High-throughput sequencing was performed using the NovaSeq6000 NGS platform (Illumina Inc.) by Genedenovo (Guangzhou, China).

### Western blotting

The protocol of Western blotting was described previously 5. The antibodies used included HNRNPA2B1 (Proteintech, Chicago, IL, USA), Flag (Proteintech), HA (Proteintech), E-cadherin (Cell Signaling Technology, Beverly, MA, USA), FOXP3 (Abcam, Cambridge, MA, USA), FAP (Proteintech), α-SMA (Proteintech), actin (Santa Cruz Biotechnology, Santa Cruz, CA, USA), Twist1 (ABclonal, Wuhan, China). The Clarity Western ECL substrate kit (Bio-Rad, Hercules, CA, USA) was used to detect the signals.

### Plasmids and transfection

The lncRNA LINC00982 5'UTR-ORF sequences were cloned into the mutant EGFP vector with the start codon ATG mutated to ATT. The mutant constructs for 5'UTR-ORFmut-GFPmut, 5'UTR-ORFmut-Flag, HNRNPA2B1-RGGmut, and *CHEK2*-m6Amut, were established by the Mut Express II Fast Mutagenesis Kit v2 (Vazyme, Nanjing, China). The siRNA against *CHEK2* (SIH600921A) was obtained from SABiosciences (Frederick, MD, USA). The siRNA against HNRNPA2B1, YY1, FOXP3, HNF1A, CEBPA, and the plasmids expressing single guide RNAs against PRDM16-DT and FOXP3, the plasmids 5'UTR-ORF-Flag, stable ORF, and ORFmut-overexpression, and plasmids expressing HNRNPA2B1, L-*CHEK2* and S-*CHEK2*, were purchased from TransheepBio (Shanghai, China). The plasmids pcDNA3.1-HNRNPA2B1-mut1-HA, pcDNA3.1-HNRNPA2B1-mut2-HA, and pcDNA3.1-HNRNPA2B1-mut3-HA were obtained by PCR amplification. The protocols for establishing stable cell lines and transfection were described previously 40. The primers for cloning, mutation, and siRNAs and sgRNAs sequences are listed in Table S1.

### Immunofluorescence staining

5'UTR-ORF or 5'UTR-ORFmut-fused GFP vectors were transfected into CRC cells, and GFP fluorescence was visualized by fluorescence microscopy (Nikon, Tokyo, Japan). As previously described 41, cells expressing 5'UTR-ORF-Flag or 5'UTR-ORFmut-Flag were fixed with 4% paraformaldehyde, permeabilized with 0.1% TrintonX-100 or 15 min, and then incubated with anti-PRDM16-DT or -Flag antibodies (Proteintech) after washing and blocking with BSA. Secondary antibodies (Proteintech) were used for staining in the dark, followed by DAPI (Beyotime Biotechnology, Shanghai, China) staining and immunofluorescence analysis.

### Generation of anti-PRDM16-DT antibody

The anti-PRDM16-DT antibody was generated by GL Biochem (Shanghai, China). Briefly, a Keyhole limpethemocyanin (KLH)-coupled peptide, TPPSTPNPAECGGDLGRRQGL-Cys, was used to produce a polyclonal antibody against PRDM16-DT by inoculating rabbits. The antibody was further purified by affinity chromatography on columns containing the KLH-coupled peptide.

### TMA and IHC analyses

A TMA (Shanghai Outdo Biotech, Shanghai, China) containing 93 CRC and 87 normal tissues was used to analyze PRDM16-DT expression. The protocol of IHC was described previously 41. The sample slides were blocked with normal serum, incubated with anti-PRDM16-DT antibody overnight at 4°C, and then incubated with the corresponding secondary antibody. 3,3'-Diaminobenzidine was used to visualize the staining. IHC staining intensity was classified as follows: Samples with a score of 1 or 2 were considered to have low expression, whereas scores of 3 or 4 were considered to have high expression.

### Invasion and migration assays

Boyden chambers were used to determine CRC metastasis ability, as previously described 18. Cells in serum-free medium were placed in the upper chamber for the migration assay, or the upper chamber was coated with Matrigel (BD Biosciences, Bedford, MA, USA) for the invasion assay. The lower chamber contained the medium with FBS. After 24 h, the migrated and invaded cells were fixed with methanol and stained with 1% crystal violet. The number of invaded cells was quantified.

### Wound healing assay

The assay was performed as described previously 40. CRC cells were plated in 12-well plates and maintained overnight. A 10 μL pipette tip was used to produce a scratch on the plates, which were then washed with PBS and cultured with serum-free medium for 24 h at 37°C. The wounds at 0 and 24 h were photographed and quantified.

### Cell viability assay

The WST-1 Cell Proliferation and Cytotoxicity Assay Kit (Beyotime Biotechnology) was used to measure cell viability, as described previously 41. CRC cells were seeded into 96-well plates. WST-1 was added to the plate and incubated with the cells for 2 h, at 37°C. Absorbance at the wavelength of 450 nm was measured using an automated microplate spectrophotometer (BioTek Instruments, Winooski, VT, USA).

### Colony-formation assay

CRC cells subjected to different treatments were seeded into 6- or 12-well plates and cultured for two weeks. The cells were then washed three times with phosphate-buffered saline (PBS), fixed with 75% ethanol for 10 min, and stained with 1% crystal violet for 10 min. Finally, the number of colonies formed was counted.

### Chromatin Immunoprecipitation (ChIP) assay

The ChIP assay was performed using the ChIP Kit (Suyan Technology Co., Ltd). Cells were collected and fixed with formaldehyde at room temperature for 10 min. Chromatin was sonicated to fragments and incubated with the FOXP3 antibody or IgG, and then ChIP-grade protein G agarose beads were added and incubated for 30 min at room temperature. After washing, DNA fragments were purified and analyzed by qPCR. The primers are listed in Table S1.

### Luciferase reporter assay

The wild-type or mutant PRDM16-DT promoter plasmids were constructed using the Mut Express II Fast Mutagenesis Kit v2 (Vazyme). In brief, CRC cells were seeded in 24-well plates and following transfection with plasmids for 48 h. The Dual-Luciferase Reporter System (Promega, Fitchburg, WI, USA) was used to analyze the Firefly and Renilla (an internal control for normalization, Promega) luciferase following the manufacturer's protocol.

### RNA-sequence, mass spectrometry, and bioinformatics analysis

Illumina HiSeq 2500 (LC Bio, Zhejiang, China) was used for RNA sequencing. DEGs were determined by DESeq. Peptide samples were analyzed by ORbitrap Fusion Lumos mass spectrometer (Thermo Fisher Scientific, San Jose, CA) as previously described 41. Then Spectronaut software (Omicsolution, Shanghai, China) was used to analyze the raw data. Pathway analysis was performed using KEGG and Gene Ontology (GO).

### Co-immunoprecipitation (Co-IP)

IP lysis buffer (Cell Signaling Technology) was used to collect the protein of CRC cells 41. Cell lysates were incubated with IgG (Santa Cruz Biotechnology) and protein A/G Sepharose beads (Invitrogen, Gaithersburg, MD, USA) at 4°C for 2 h, and the supernatant was collected and incubated with primary antibodies overnight at 4°C. Then A/G Sepharose beads were added and incubated for 4 h at 4°C. Beads were collected and washed with PBS and IP lysis buffer three times, then mixed with loading buffer for Western blotting.

### QRT-PCR and RT-PCR

As previously described 40, total RNA of CRC cells was isolated using TRIzol total RNA isolation reagent (Invitrogen). Reverse transcription was performed using the PrimeScript™ II First Strand cDNA Synthesis Kit (Takara, Dalian, China). SYBR® Premix Ex Taq™ II (Takara) was used to analyze the mRNA expression of LINC00982, *CHEK2*, and the internal control, GAPDH, using a MiniOpticon™ RT-PCR system (Bio-Rad). The primers used are listed in Table S1.

### Multiplex immunohistochemical staining

The PANO 6-plex IHC Kit (Panovue, Beijing, China) was used to analyze FOXP3, PRDM16-DT, and HNRNPA2B1 expression and their correlation with clinicopathological parameters in a TMA. Briefly, the slides were deparaffinized in xylene, rehydrated in ethanol, blocked with the blocking buffer (Panovue), incubated with primary antibodies for 60 min, washed with the TBST buffer, and incubated with horseradish peroxidase-conjugated secondary antibodies for 10 min. The slides were incubated with primary antibodies and visualized using Opal™ 520 TSA (1:200 dilution), followed by DAPI staining for 10 min. A Vectra Polaris automated pathology imaging platform (PerkinElmer, Waltham, MA, USA) was used to acquire images, which were analyzed using the HALO digital pathology analysis platform (Indica Labs, Corrales, NM, USA).

### RNA affinity purification

RNA pulldown Kit (Suyan Technology Co., Ltd, Guangzhou, China) was used to perform the RNA affinity assay. Briefly, the biotinylated RNA, synthesized by RiboBio (Guangzhou, China), was incubated with streptavidin magnetic beads and cell lysates at 4 °C overnight. After washing and eluting, the mixture was subjected to Western blotting. The biotin-labeled RNAs used are listed in Table S1.

### RNA immunoprecipitation (RIP) assay

The interactions between HNRNPA2B1 and exon 9 of *CHEK2* were analyzed by an RNA Immunoprecipitation (RIP) kit (Suyan Technology Co., Ltd). Cells were collected, lysed, and incubated with the RIP buffer containing magnetic beads conjugated to the HNRNPA2B1 antibody or IgG (negative control). The RNA was extracted from the sample and analyzed by qRT-PCR.

### MeRIP-quantitative PCR

MeRIP was analyzed using the BiboMeRIP^TM^ m6A Transcriptome Profiling Kit (Ribobio, Guangzhou, China). RNA fragmentation buffer was used to fragment the mRNA into 100-nucleotide fragments. Subsequently, the fragments were incubated with the m6A antibody for 2 h at 4°C. Finally, one-step RT-PCR (RT-PCR; Bio-Rad) was performed to detect m6A-modified target genes (Table S1).

### Fluorescence *in situ* hybridization (FISH)

FITC-labeled probes were synthesized by RiboBio (Guangzhou, China). The Fluorescence *in situ* Hybridization Kit (RiboBio) was used according to manufacturer's protocol to observe the probe signals. Images were acquired by laser scanning confocal microscopy (Carl Zeiss AG, Jena, Thuringia, German).

### Single-cell gel electrophoresis

Comet assay was performed according to manufacturer's protocol (Trevigen, MD, USA). Briefly, cells were collected and mixed with melt agarose at 1:10 ratio, then spread on pre-warmed glass slides. The slides were immersed in the lysis solution (Trevigen) for 1 h, placed in 1× neutral electrophoresis buffer for 30 min, and electrophoresed for 45 min at 4°C. Cells were fixed and stained with SYBR™ Gold (Invitrogen). Tail length and intensity of DNA in the tail was quantified.

### ELISA

E-Cadherin ELISA kit (Abcam, Cambridge, MA, USA) was used according to the manufacturer's protocol. Briefly, CRC cells rinsed by PBS buffer and subsequently treated with 500 μL fresh complete medium for 48 h. The supernatant was collected by centrifugation (300 ×g, 10 min), and the level of secreted E-Cadherin in the conditioned medium (CM) was measured.

### Molecular docking

The FOXP3 protein model was constructed using the protein databank (PDB; http://www.rcsb.org). The pose with the best cimicifugoside H-1 and FOXP3 binding score was selected for interaction analysis using Dock version 6.9 (https://dock.compbio.ucsf.edu/).

### Purification of FOXP3 protein

Plasmid pET-28b-FOXP3 was constructed and transformed into E. coli BL21 (DE3) star cells, as described previously 41. When the optical density of the bacterial culture reached about 0.6 at 600 nm, 0.5 mM isopropyl β-D-thiogalactopyranoside was added to the culture and incubated for 12 h, at 37°C. A His-tag protein purification kit (Beyotime Biotechnology) was used to isolate the His-tagged fusion protein.

### Biacore assay

The Biacore X100 system (GE Healthcare Life Sciences, USA) was used to analyze the binding of FOXP3 and cimicifugoside H-1 according to the manufacturer's instructions, as previously described 41. Briefly, purified protein was coupled with CM7 chip (GE Healthcare Life Sciences). Different concentrations of cimicifugoside H-1 were loaded to detect the response values. Biacore analysis software was used to analyze the Kd value of FOXP3 and cimicifugoside H-1.

### Establishment of OR CRC Sublines

CRC HCT116-Luc cells were injected intravenously into mice through the tail vein. One week after cell injection, the mice received oral gavage of oxaliplatin (40 mg/kg) every two days for three weeks. Then, HCT116-Luc-OR cells that invaded the lungs were isolated and cultured. The IC50 of HCT116-Luc and HCT116-Luc-OR were measured by the WST-1 Cell Proliferation and Cytotoxicity Assay Kit (Beyotime Biotechnology), as previously described 5.

### *In vivo* experimental metastasis model

Female NOD-Prkdc^em26Cd52^Il2rg^em26Cd22^ (NCG) mice aged 6-8 weeks were maintained following the institutional guidelines. Cells suspended in PBS were injected intravenously into mice through the tail vein. Mice were fed either DOX-containing or a normal diet. Four weeks after cell injection, IVIS Lumina III imaging system (PerkinElmer, Hopkinton, MA, USA) was used to observe metastasis by bioluminescence imaging. In the drug treatment experiments, CRC cells were intravenously injected into the tail vein of mice and orally administrated with vehicle (0.5% CMC-Na), cimicifugoside H-1 (20 mg/kg), or oxaliplatin (20 mg/kg) every two days for three weeks, and lung metastasis was monitored.

### Statistical analysis

All *in vitro* experiments were performed three times. The GraphPad Prism software was used for two-tailed Student's tests, one-way ANOVA analysis, two-way ANOVA, and log-rank test analysis. The values were presented as the mean ± SD. Survival was analyzed by Kaplan-Meier method and log-rank test. Bars, SD; *P < 0.05; **P < 0.01; ***P < 0.001; ns, no significant difference.

## Supplementary Material

Supplementary figures and table 1.

Supplementary table 2: DIA-based proteomics.

Supplementary table 3: DIA-based proteomics.

## Abbreviations

CRC: colorectal cancer
LncRNA: long-noncoding RNA
AS: alternative splicing
KEGG: kyoto encyclopedia of genes and genomes
OR: oxaliplatin resistant
DIA: data-independent acquisition
DEPs: differentially expressed proteins
DOX: doxycycline
CPT: camptothecin
ChIP: chromatin Immunoprecipitation
Co-IP: co-immunoprecipitation
RIP: RNA immunoprecipitation
FISH: fluorescence *in situ* hybridization
