# Supplementary material for: LINC00982-encoded protein PRDM16-DT regulates CHEK2 splicing to suppress colorectal cancer metastasis and chemoresistance
Source: Theranostics. 2024 May 27;14(8):3317–38. doi: 10.7150/thno.95485 (PMC11155395; doi:10.7150/thno.95485)
Supplement: Supplementary file 1 — Supplementary figures and table 1. [file thnov14p3317s1.pdf]

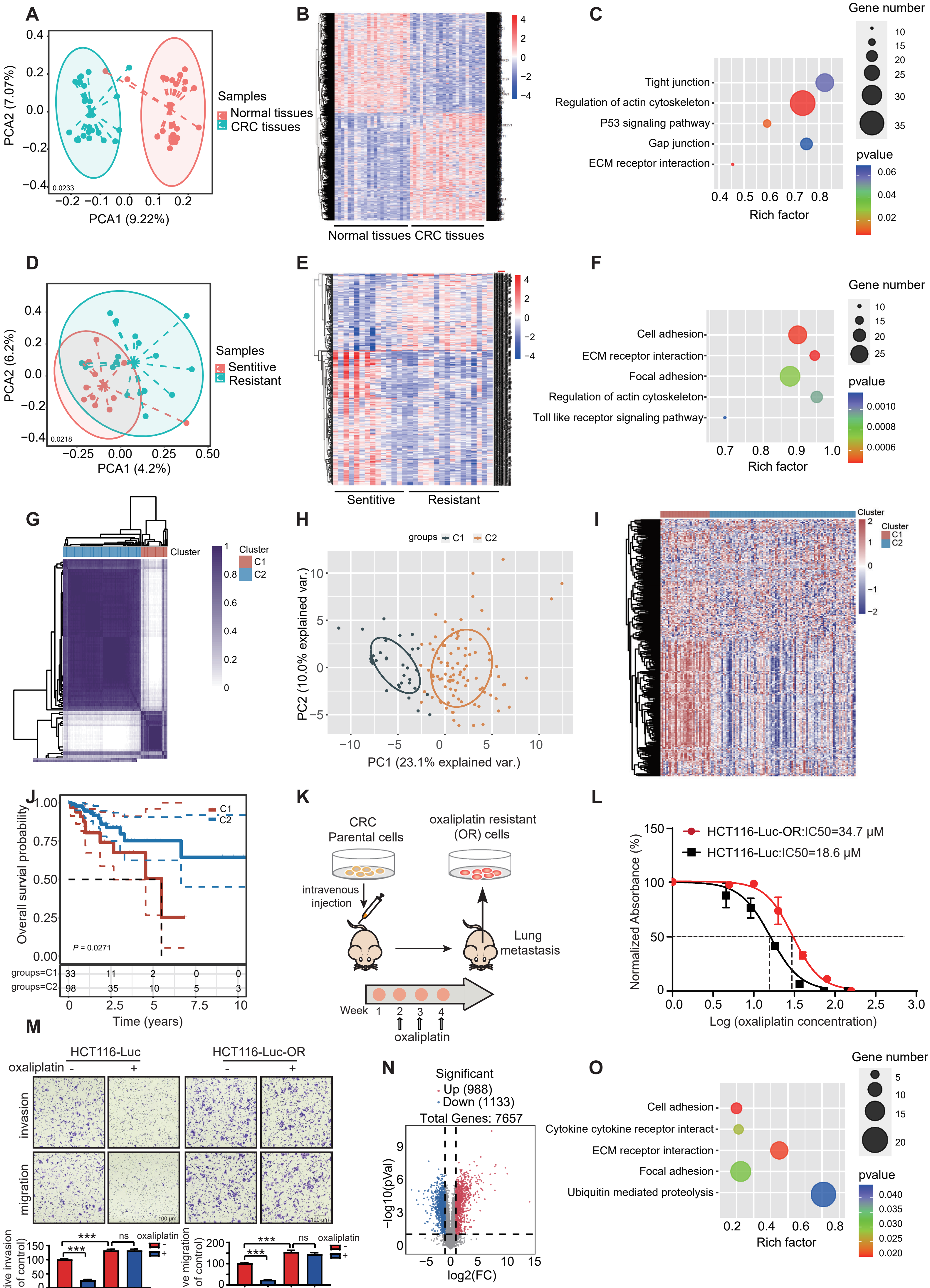
A
PCA2 (7.07%)
PCA1 (9.22%)
Samples
Normal tissues
CRC tissues
0.0233
B
Normal tissues CRC tissues
C
Tight junction
Regulation of actin cytoskeleton
P53 signaling pathway
Gap junction
ECM receptor interaction
Rich factor
Gene number
pvalue
D
PCA2 (6.2%)
PCA1 (4.2%)
Samples
Sentitive
Resistant
0.0218
E
Sentitive
Resistant
F
Cell adhesion
ECM receptor interaction
Focal adhesion
Regulation of actin cytoskeleton
Toll like receptor signaling pathway
Rich factor
Gene number
pvalue
G
Cluster
C1
C2
H
groups C1 C2
PC2 (10.0% explained var.)
PC1 (23.1% explained var.)
I
Cluster
C1
C2
J
Overall survival probability
C1
C2
P = 0.0271
groups=C1 33 11 2 0 0
groups=C2 98 35 10 5 3
Time (years)
K
CRC
Parental cells
oxaliplatin resistant
(OR) cells
intravenous
injection
Lung
metastasis
Week 1 2 3 4
oxaliplatin
L
HCT116-Luc-OR:IC50=34.7 μM
HCT116-Luc:IC50=18.6 μM
Normalized Absorbance (%)
Log (oxaliplatin concentration)
M
HCT116-Luc
HCT116-Luc-OR
oxaliplatin - + - +
invasion
migration
100 μm
Relative invasion (% of control)
Relative migration (% of control)
HCT116-Luc HCT116-Luc-OR
ns
N
Significant
Up (988)
Down (1133)
Total Genes: 7657
−log10(pVal)
log2(FC)
O
Cell adhesion
Cytokine cytokine receptor interact
ECM receptor interaction
Focal adhesion
Ubiquitin mediated proteolysis
Rich factor
Gene number
pvalue

Figure S1

Figure S1. Identification of key drivers of cancer progression and chemoresistance.

(A) Principal component analysis of GEO dataset (GSE52056), including 23 CRC and paired normal tissues. (B) Hierarchical clustering analysis of DEGs between CRC and paired normal tissues; fold-change ⩾ 1.5, p-value ⩽ 0.05. (C) KEGG analysis of the DEPs between CRC and paired normal tissues. (D) Principal component analysis of GEO dataset (GSE69657), including 13 cases of chemotherapy-sensitive and 17 cases of chemotherapy-resistant CRC tissues. (E) Hierarchical clustering analysis of DEGs in chemoresistance of CRC; fold-change ⩾ 1.5, p-value ⩽ 0.05. (F) KEGG analysis of key pathways in CRC chemoresistance. (G) 131 CRC tissues with chemotherapy clustered into two subtypes (C1 and C2). (H) Principal component analysis of 131 CRC tissues with chemotherapy. (I) Heatmap features of the two clusters (C1 and C2). (J) Longer overall survival of C2 than C1. (K) HCT116-Luc cells were intravenously injected into the tail vein of mice and orally administrated with oxaliplatin (40 mg/kg). After three weeks, lung metastasis was monitored, and invaded cells (HCT116-Luc-OR) were isolated and cultured. (L) HCT116-Luc-OR showed high resistance to oxaliplatin-mediated inhibitory effects on cell growth (IC50: 34.7 μM). n = 3/experiments. (M) OR CRC cells were treated with oxaliplatin (20 μM) for 48 h, and the metastasis ability analyzed. n = 3/experiments; Scale bar, 100 μm. (N) Volcano plot showing DEPs in OR cells. A total of 477 DEPs (fold-change⩾1.5, p-value⩽0.05) were identified, including 1133 downregulated and 988 upregulated proteins. n = 3/experiments. (O) KEGG analysis of the key pathways in chemoresistance of CRC. Bars, SD; **, P < 0.01; ***, P < 0.001

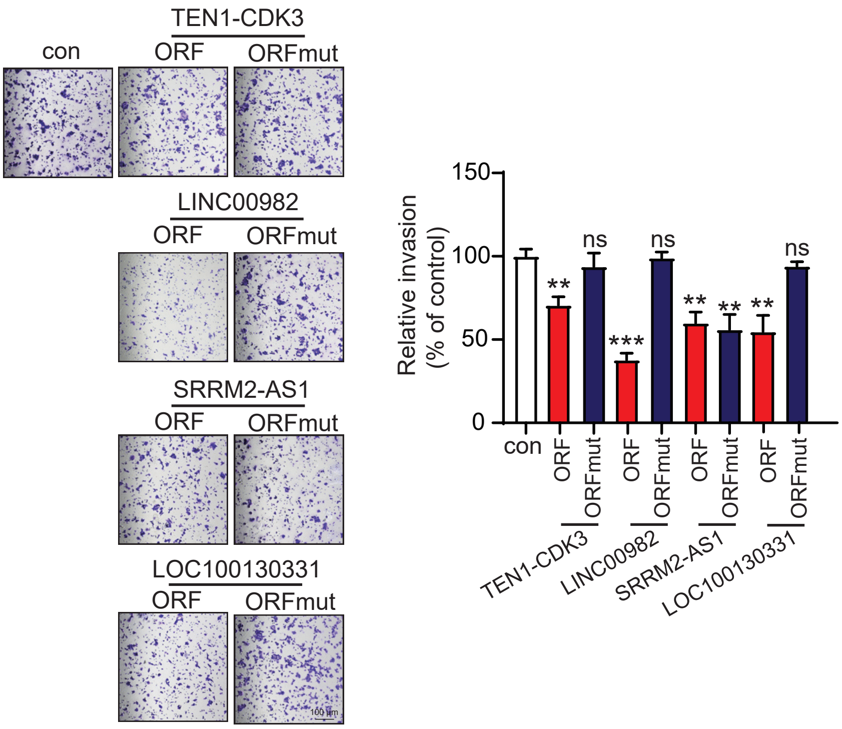

Figure S2

Figure S2. PRDM16-DT encoded by LINC00982 exerts an inhibitory effect on CRC invasion. n = 3/experiments. Scale bar, 100 µm. Bars, SD; **, $P < 0.01$; ***, $P < 0.001$; ns, no significant difference.

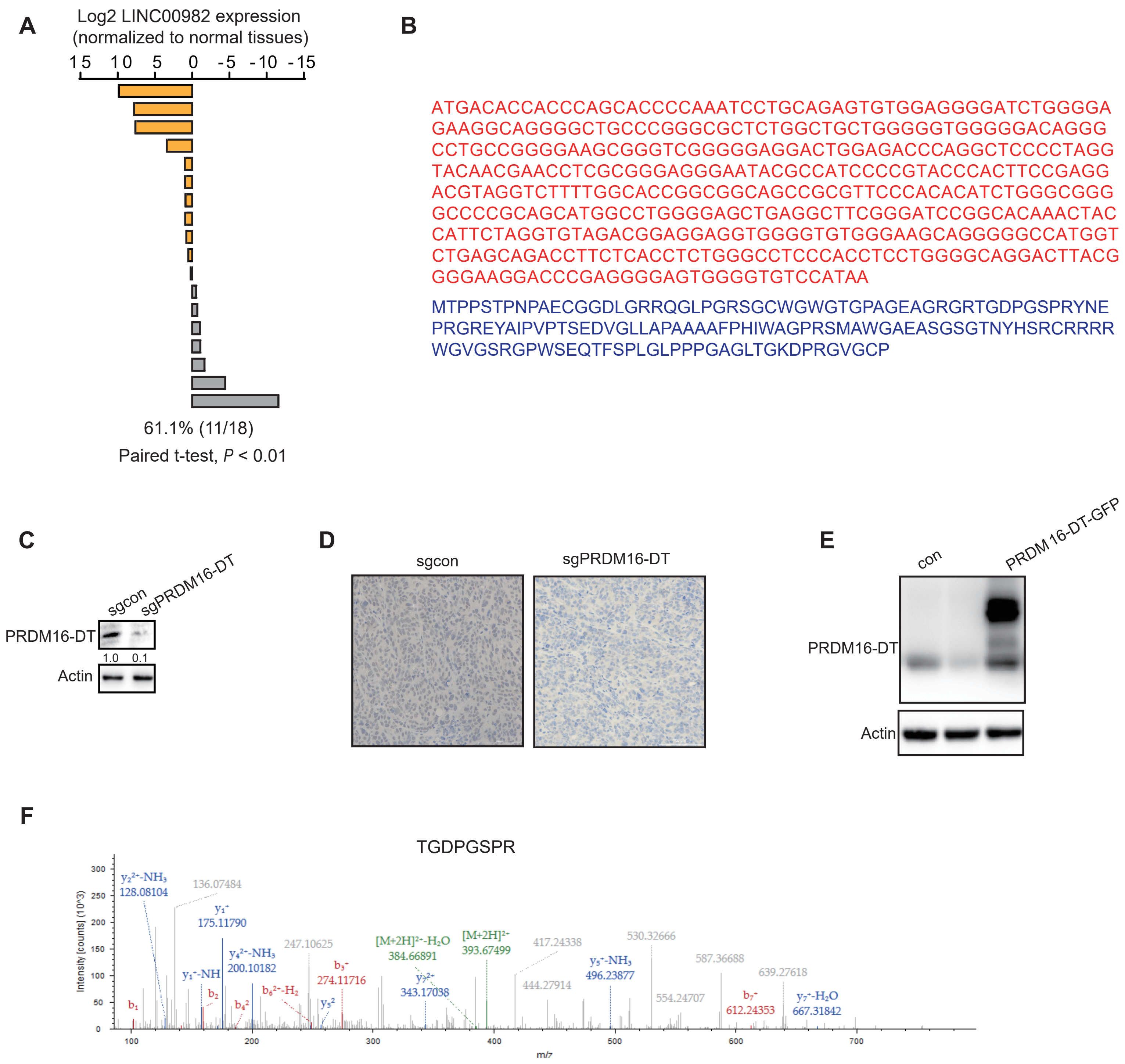

Figure S3

Figure S3. lncRNA LINC00982 and PRDM16-DT expression is downregulated in CRC tissues. (A) Lower expression level of LINC00982 in CRC tissues compared to normal tissues. (B) LINC00982 contains a potential small protein-encoding ORF. (C) Successful establishment of PRDM16-DT-deficient CRC cells. (D) PRDM16-DT staining in tumors derived from PRDM16-DT-deficient and control cells. n = 3/experiments. Scale bar, 100 μm. (E) PRDM16-DT expression was detected by Western blotting assay. n = 3/experiments. (F) A unique peptide was identified using mass spectrometry. n = 3/experiments. Bars, SD; **, P < 0.01; ***, P < 0.001.

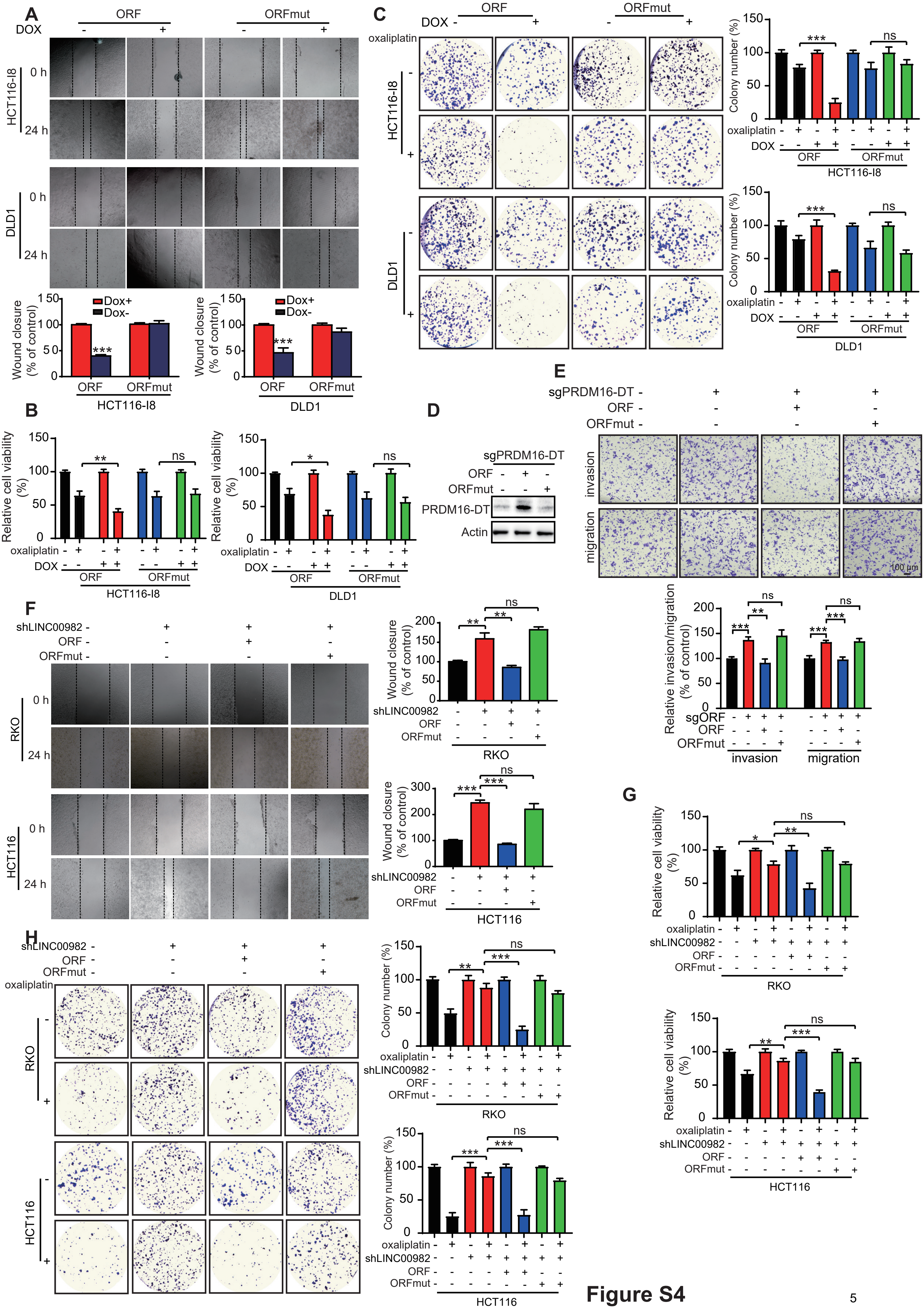
A
ORF
ORFmut
DOX
HCT116-I8
DLD1
0 h
24 h
Wound closure (% of control)
Dox+
Dox-
***
ORF
ORFmut
HCT116-I8
DLD1
B
Relative cell viability (%)
oxaliplatin
DOX
**
*
ns
C
oxaliplatin
Colony number (%)
***
ns
D
sgPRDM16-DT
ORF
ORFmut
PRDM16-DT
Actin
E
sgPRDM16-DT
invasion
migration
100 μm
Relative invasion/migration (% of control)
sgORF
F
shLINC00982
RKO
HCT116
G
H
oxaliplatin

Figure S4

Figure S4. PRDM16-DT protein, not its lncRNA, suppresses CRC cell migration and reverses oxaliplatin resistance.

(A) Wound healing assay showed that ORF significantly suppressed CRC migration ability and cell viability. Cells engineered for DOX-inducible expression of PRDM16-DT were treated with DOX (1 mg/mL) for 48 hours. n = 3/experiments. (B-C) WST-1 and colony-forming assays showed that PRDM16-DT overexpression significantly enhanced the anticancer effects of oxaliplatin (20 μM). n = 3/experiments. (D-E) ORF or ORFmut were re-overexpressed in PRDM16-DT-deficient cells (D), and metastasis assay was performed to compare their metastatic ability (E), n = 3/experiments. Scale bar, 100 μm. (F) Wound healing assay showed that knockdown of LINC00982 markedly promoted the CRC migration ability, and restored expression with ORF significantly abrogated the promotion effect of silencing LINC00982. n = 3/experiments. (G-H) WST-1 and colony-forming assays showed the oxaliplatin resistance of PRDM16-DT-knockdown cells transfected with ORF or ORFmut plasmids and treated with oxaliplatin (20 μM). n = 3/experiments. Bars, SD; **, $P < 0.01$; ***, $P < 0.001$; ns, no significant difference.

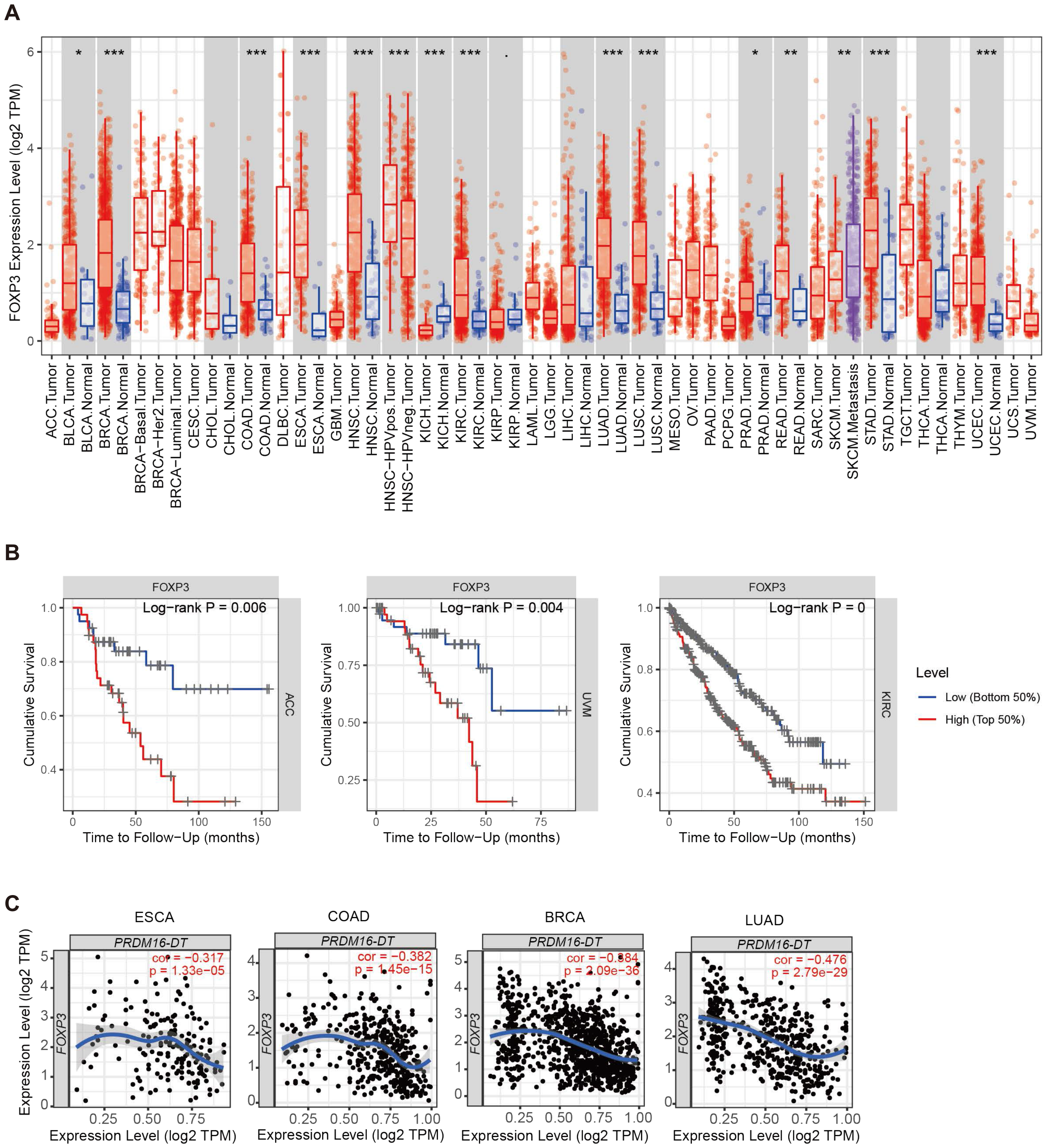
A
FOXP3 Expression Level (log2 TPM)
B
FOXP3
Log−rank P = 0.006
Cumulative Survival
Time to Follow−Up (months)
ACC
Log−rank P = 0.004
UVM
Log−rank P = 0
KIRC
Level
Low (Bottom 50%)
High (Top 50%)
C
ESCA
COAD
BRCA
LUAD
PRDM16-DT
cor = −0.317
p = 1.33e−05
cor = −0.382
p = 1.45e−15
cor = −0.384
p = 2.09e−36
cor = −0.476
p = 2.79e−29
FOXP3
Expression Level (log2 TPM)

Figure S5

Figure S5. FOXP3 expression is of clinical significance in cancers.

(A) FOXP3 expression levels in various cancers analyzed using TIMER. Most tumor tissues showed higher FOXP3 expression. (B) Based on FOXP3 expression from the TIMER database, Kaplan-Meier plot analysis showed that patients with high expression of FOXP3 had shorter survival than those with low FOXP3 expression. (C) Reciprocal correlation between FOXP3 and PRDM16-DT mRNA expression in ESCA, COAD, BRCA, and LUAD was analyzed using Tumor Immune Estimation Resource (TIMER, cistrome.shinyapps.io/timer). Bars, SD; **, $P < 0.01$; ***, $P < 0.001$.

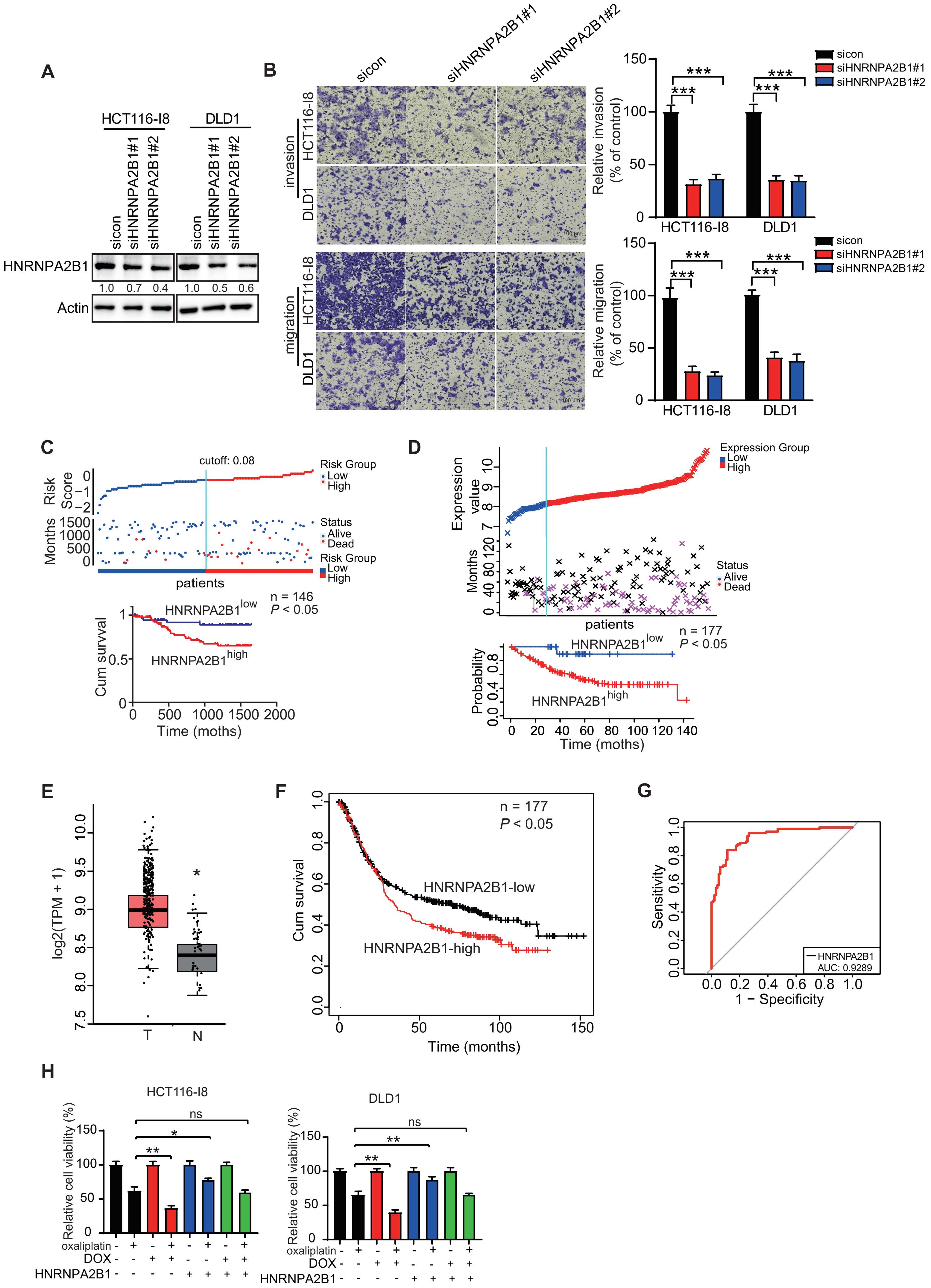
A
B
C
D
E
F
G
H
HCT116-I8
DLD1
sicon
siHNRNPA2B1#1
siHNRNPA2B1#2
HNRNPA2B1
Actin
1.0 0.7 0.4 1.0 0.5 0.6
invasion
migration
Relative invasion (% of control)
Relative migration (% of control)
cutoff: 0.08
Risk Score
Risk Group
Low
High
Status
Alive
Dead
Months
patients
n = 146
P < 0.05
Cum survival
Time (moths)
Expression value
Expression Group
n = 177
Probability
log2(TPM + 1)
T
N
HNRNPA2B1-low
HNRNPA2B1-high
Time (months)
Sensitivity
1 − Specificity
HNRNPA2B1
AUC: 0.9289
Relative cell viability (%)
oxaliplatin
DOX
ns

Figure S6

Figure S6. HNRNPA2B1 expression contributes to tumorigenesis and correlates with poor prognosis in CRC.

(A) Successful knockout of HNRNPA2B1 in CRC cells. n = 3/experiments. (B) HNRNPA2B1 knockdown reduced HCT116-I8 and DLD1 cell migration and invasion. n = 3/experiments. Scale bar, 100 μm. (C) Distribution of risk scores along with survival statuses and HNRNPA2B1 expression profiles for CRC. (D) Kaplan-Meier survival analysis based on GSE20916 and GSE17536 revealed significantly shorter survival in patients with high HNRNPA2B1 expression than those with low HNRNPA2B1 expression. (E-F) Analysis of the TCGA data showing higher HNRNPA2B1 expression in tumor tissues than in normal tissues (E). Survival of colorectal cancer patients was analyzed using the Gene Expression Profiling Interactive Analysis (GEPIA) database (F). (G) Receiver operating characteristic (ROC) curve. (H) HNRNPA2B1 overexpression significantly abolished the inhibitory effect of PRDM16-DT on oxaliplatin resistance. n = 3/experiments. Bars, SD; **, $P < 0.01$; ***, $P < 0.001$; ns, no significant difference.

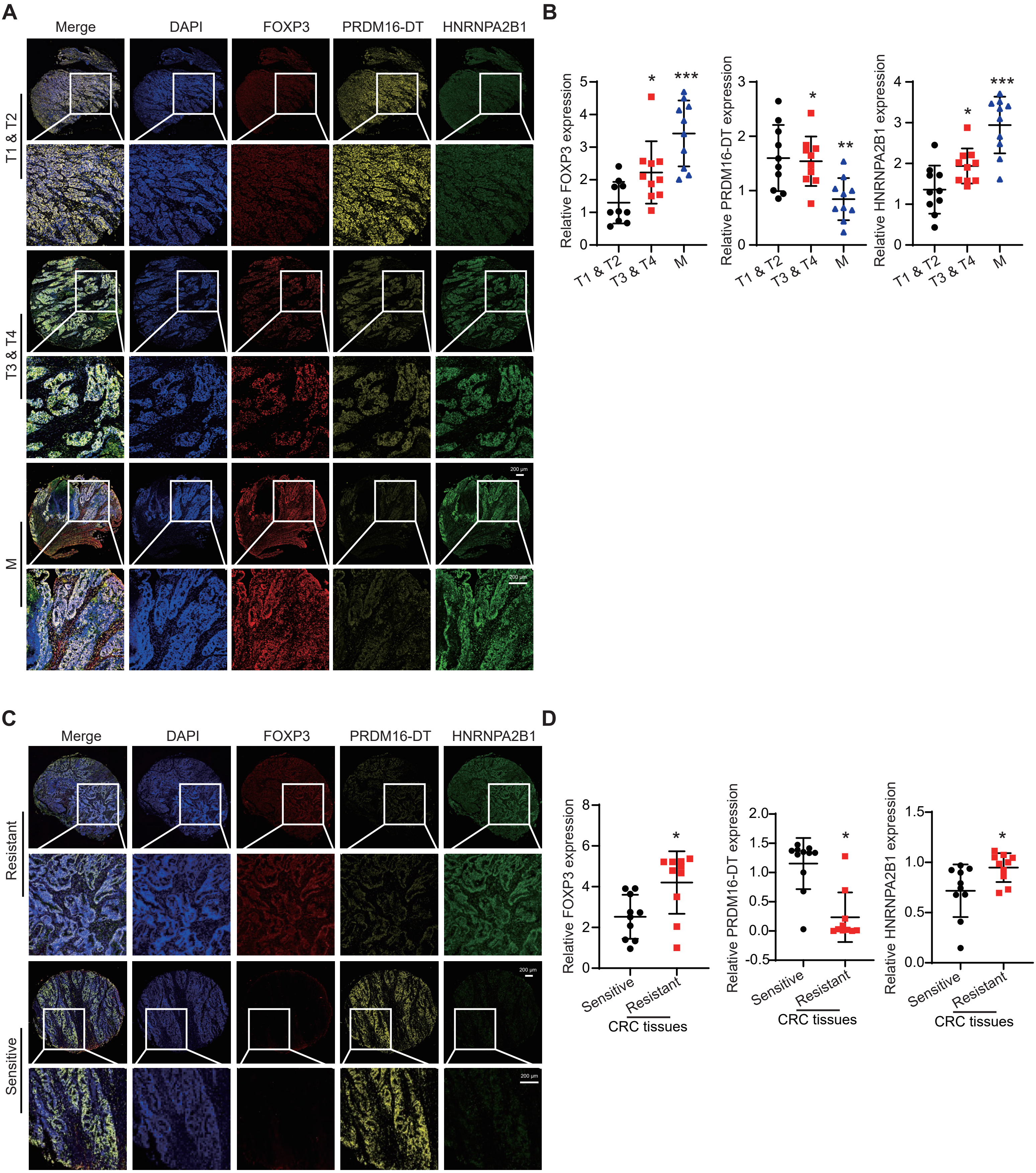
A
Merge
DAPI
FOXP3
PRDM16-DT
HNRNPA2B1
T1 & T2
T3 & T4
M
200 μm
B
Relative FOXP3 expression
Relative PRDM16-DT expression
Relative HNRNPA2B1 expression
T1 & T2
T3 & T4
M
C
Resistant
Sensitive
D
Sensitive
Resistant
CRC tissues

Figure S7

Figure S7. FOXP3/PRDM16-DT/HNRNPA2B1 regulatory axis is of clinical significance in cancers.

(A) Representative immunohistochemistry images of FOXP3, PRDM16-DT, and HNRNPA2B1 in primary (n = 20) and metastatic (n = 10) CRC tissues. (B) Comparison of FOXP3, PRDM16-DT, and HNRNPA2B1 expression in primary tumors and normal tissues. (C) Representative immunohistochemistry images of FOXP3, PRDM16-DT, and HNRNPA2B1 in oxaliplatin-sensitive (n = 10) and oxaliplatin-resistant CRC (n = 10) tissues. (D) Comparison of FOXP3, PRDM16-DT, and HNRNPA2B1 expression in 10 cases of OR CRC tissues and 10 cases of oxaliplatin-sensitive CRC tissue. Bars, SD; **, $P < 0.01$; ***, $P < 0.001$; ns, no significant difference.

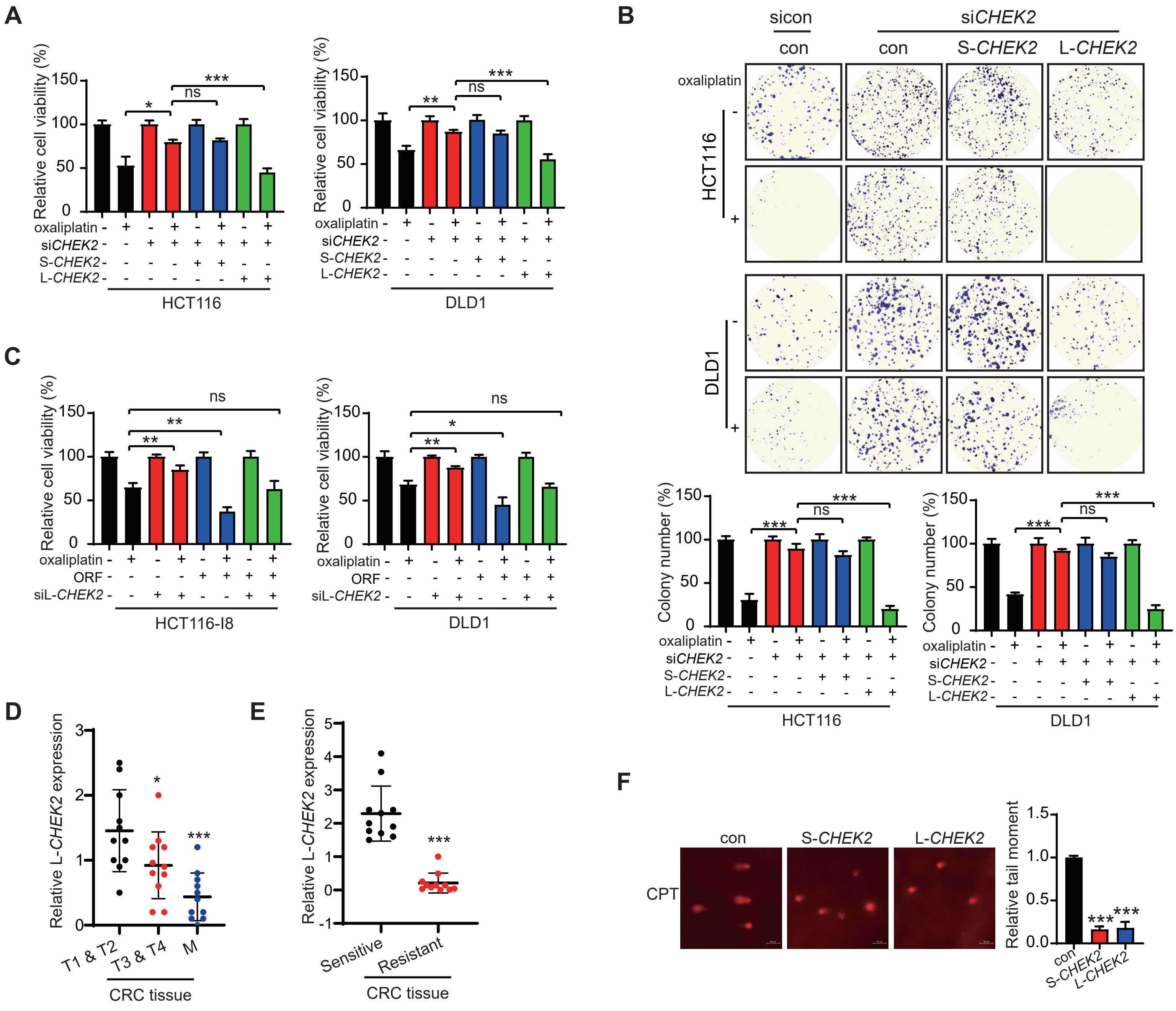

Figure S8

Figure S8. L-*CHEK2* is of clinical significance in oxaliplatin resistance.

(A-B) WST-1 and colony-forming assays showed that L-*CHEK2* overexpression significantly enhanced the anticancer effects of oxaliplatin (20 μM). n = 3/experiments. (C) L-*CHEK2* knockdown significantly abolished the inhibitory effect of PRDM16-DT on oxaliplatin resistance. n = 3/experiments. (D) Comparison of L-*CHEK2* mRNA expression in primary tumors (T1 & 2, T3 & 4) (n = 20) and metastatic tumors (M) (n = 10). (E) Comparison of L-*CHEK2* mRNA expression in oxaliplatin-sensitive (n = 10) and oxaliplatin-resistant CRC (n = 10) tissues. (F) Representative images and quantification of damaged DNA in indicated cells analyzed by comet assay. The indicated cells were treated with CPT (10 μM). n = 3/experiments. bar, 50 μm. Bars, SD; **, $P < 0.01$; ***, $P < 0.001$; ns, no significant difference.

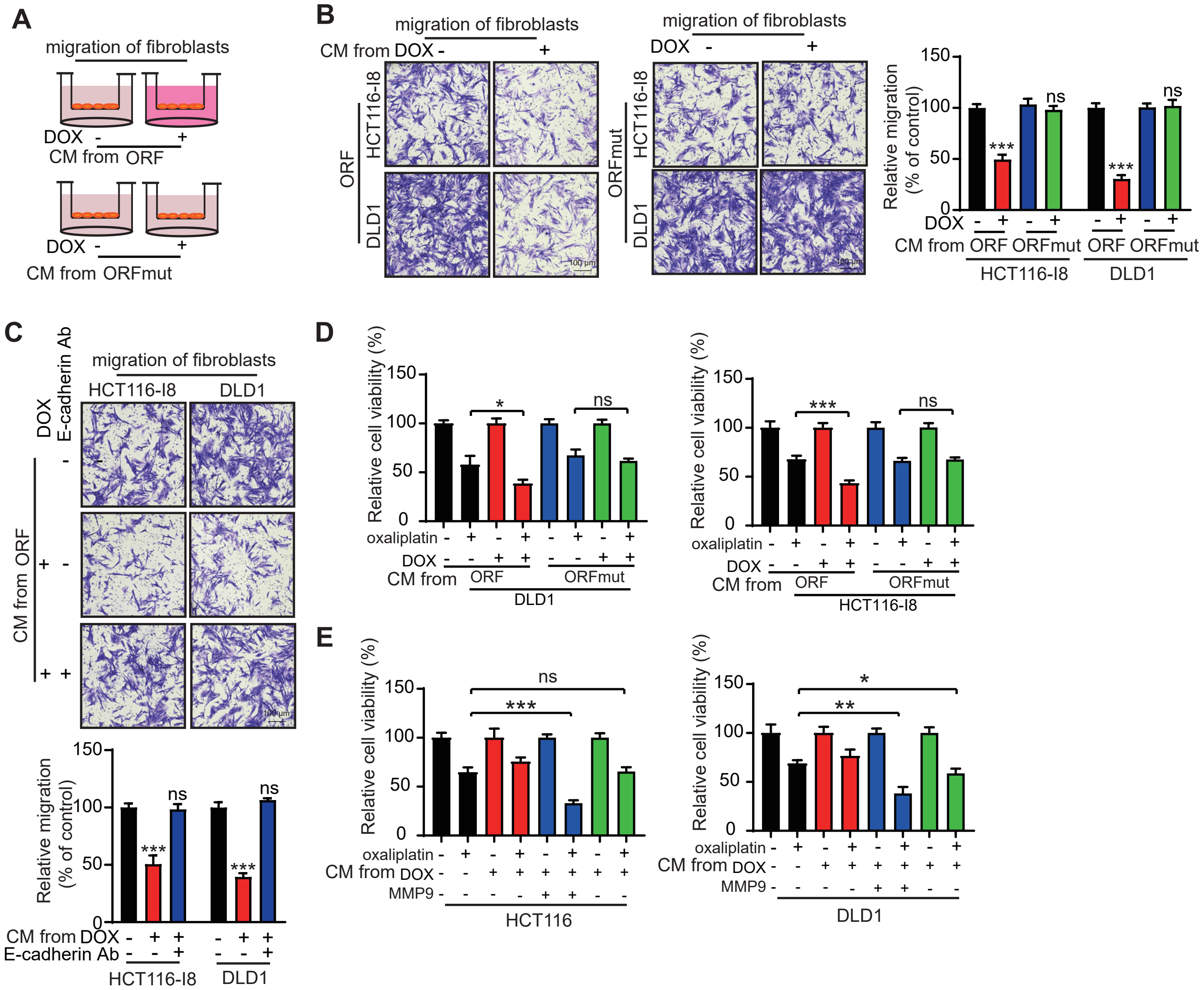

Figure S9

Figure S9. PRDM16-DT reverse oxaliplatin resistance by promoting E-cadherin secretion to inhibit fibroblasts activation in a paracrine manner.

(A) Schematic diagram for coculture system. (B) Metastasis assay showed that CM from ORF-overexpressing CRC cells reduced the fibroblasts metastasis. n = 3/experiments. (C) Blockade of E-cadherin attenuated the migration of fibroblasts induced by PRDM16-DT. n = 3/experiments. (D) The oxaliplatin resistance of CRC cells treated with CM from different fibroblasts as indicated by the WST-1 assay. n = 3/experiments. (E) WST-1 assay showing that MMP9 overexpression in fibroblasts and reversal of the inhibitory effect of fibroblasts cultured with CM from ORF-overexpressing CRC cells on the oxaliplatin resistance of CRC cells as indicated. n = 3/experiments. Bars, SD; **, P < 0.01; ***, P < 0.001.

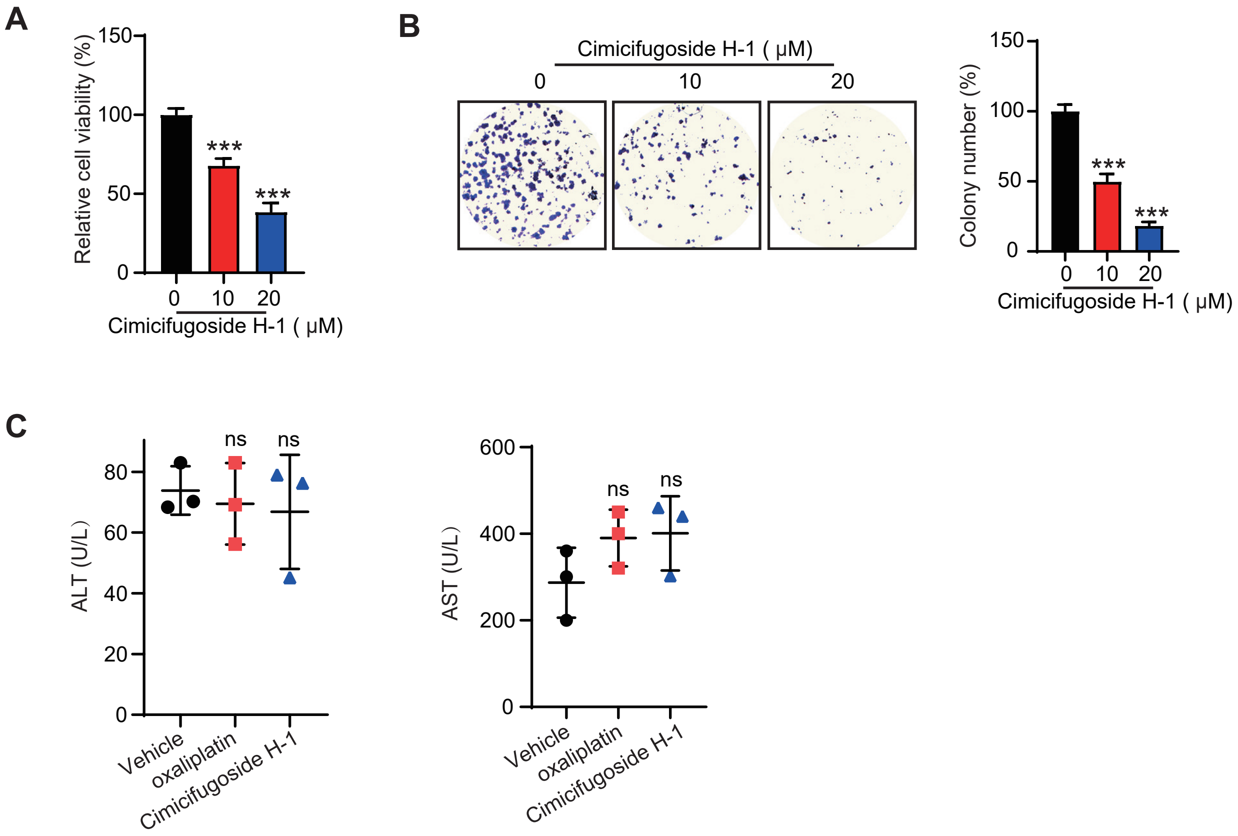

Figure S10

Figure S10. Cimicifugoside H-1 suppresses cell viability of OR cells and inhibits CRC metastasis *in vivo* with no significant side effects.

(A-B) OR cells treated with cimicifugoside H-1(10 μM) for 24 h, and the cell viability was compared. n = 3/experiments. (B) Levels of serum alanine and aspartate aminotransferase showed no significant differences in mice in the treatment and control groups. n = 3 mice/group. Bars, SD; **, P < 0.01; ***, P < 0.001; ns, no significant difference.

The sequences of primers for cloning, mutation and ChIP

ORFmut-GFPmut-up/ ORFmut-Flag-up	CCTGAGATtACACCACCCAGCACCCCAAATCC
ORFmut-GFPmut-dw/ORFmut-Flag-dw	GGTGGTGTaATCTCAGGTTCGAGTCTGCGCGA
GFPmut-up	GCCACCATtGTGAGCAAGGGCGAGGAGCTGTT
GFPmut-dw	TTGCTCACaATGGTGGCGACCGGTGGATCCCG
CHEK2mut-up	AGAAGAGGgCTGTCTTATAAAGATTACTGATTTTGGGC
CHEK2mut-dw	TAAGACAGcCCTCTTCTTGAGATGACAGTAAAACAT
HNRNPA2B1-MUT1-HA-up	TAGTCCAGTGTGGTGGAATTCATGTACCCATACGACGTGCCTG
HNRNPA2B1-MUT1-HA -dw	TGCTGGATATCTGCAGAATTCTCATTCTTTAATTCCGCCAACA
HNRNPA2B1-MUT2-HA -up	TAGTCCAGTGTGGTGGAATTCATGTACCCATACGACGTGCCTG
HNRNPA2B1-MUT2-HA -dw	TGCTGGATATCTGCAGAATTCTCAACTCCTAGAACTCTGAACTTCCTG
HNRNPA2B1-MUT3-HA -up	TAGTCCAGTGTGGTGGAATTCATGTACCCATACGACGTGCCTG
HNRNPA2B1-MUT3-HA -dw	TGCTGGATATCTGCAGAATTCTCAGTATCGGCTCCTCCCACC
HNRNPA2B1-RGG(1+2)mut-up	CAACTTTGGCTTTGGGGATTCAGCTGGTGGCGGTGGAAATTTC
HNRNPA2B1-RGG(1+2)mut-dw	TCCCCAAAGCCAAAGTTGCCTCCTGCTCCACTCCTAGAACTCTGAACTTCC
HNRNPA2B1-RGG(1+2+3)mut-up	ACTTTGCAGGAGGATCTGATGGATATGGCA
HNRNPA2B1-RGG(1+2+3)mut-dw	CAGATCCTCCTGCAAAGTTACTTCCTGGTCCTGGTCC
HNRNPA2B1-RGG(1+2+3+4)mut-up	AGGAGGAGCAGGAGGATATGGTGGTGGAGG
HNRNPA2B1-RGG(1+2+3+4)mut-dw	TATCCTCCTGCTCCTCCTCCATAACCGGGG
PRDM16-DT-PGL3-up	ATCTGCGATCTAAGTAAGCTTGGAATCACACAAAGCGTGACCTTTTATG

PRDM16-DT-PGL3-dw	CAGTACCGGAATGCCAAGCTTGGAATGAAGAGCTGACGCTTGTTACAGC
ChIP-1-up	GGAATCACACAAAGCGTGACCTTTATG
ChIP-1-dw	GGAATGAAGAGCTGACGCTTGTTACAGC
ChIP-2-up	TTTTCATGGCGACATAGTATTCCATTGCATG
ChIP-2-dw	CTTCACCAAATTGAAATGAGTGAGGAGCATT
ChIP-3-up	AGGTTTAACAGTCTTTTGATCTCAGACTT
ChIP-3-dw	CCACAGAGCCAAATGGTGAAAATGG
ChIP-4-up	ACTGGCCCAGGGAGAAACCTTTCTGA
ChIP-4-dw	TGTGCTTTTTGCTAAATAGGAAAAAC
ChIP-5-up	GCATTTCTGCAAGAAACTGGTATTTCT
ChIP-5-dw	AGTGTGCACTAAGCCTGGACCACAT

The sequences of siRNA

YY1	CCAAACAACUGGCAGAAUUTT
FOXP3	GCACAUUCCCAGAGUUCCUTT
HNF1A	CCTCCATCTAACATTCAATT
CEBPA	AUG ACA AGC UUU CCA AAU AUUTT
IRF-2	GGACCAACAAGGGCAGTGGTT
HNRNPA2B1#1	GGAGAGTAGTTGAGCCAAA
HNRNPA2B1#2	GCTACGGAGGTGGTTATGA

RT-PCR

GAPDH-up	GAGTCCACTGGCGTCTTC
GAPDH-dw	GGGGTGCTAAGCAGTTGGT
CHEK2-up	GGAATTGATGGAAGGGGGAGAGCT
CHEK2-dw	AGGCAGATAAAAAGAATAACTCC
exon9-up	GCAGTACCTTCATGAAAACGGTA
exon9-dw	AATCAGTAATCTTTATAAGACAGTCCT

The sequences of primers for qRT-PCR

LINC00982-up	CCATTCTAGGTGTAGACGGAGGAG
LINC00982-dw	GCCCAGAGGTGAGAAGGTC
GAPDH-up	CTCTGCTCCTCCTGTTCGAC
GAPDH-dw	GCGCCCAATACGACCAAATC

The sequences of fish

CHEK2-exon9-FAM	AAAAUGACAGUAGAGUUCUUCUCCUGACAG

The sequences of sgRNA

FOXP3	CCTACTTAGGCACTGCCAGG
PRDM16-DT	GTTCCCACACATCTGGGCG
